# Distinct Gene Number-Genome Size Relationships for Eukaryotes and Non-Eukaryotes: Gene Content Estimation for Dinoflagellate Genomes

**DOI:** 10.1371/journal.pone.0006978

**Published:** 2009-09-14

**Authors:** Yubo Hou, Senjie Lin

**Affiliations:** Department of Marine Sciences, University of Connecticut, Groton, Connecticut, United States of America; University of British Columbia, Canada

## Abstract

The ability to predict gene content is highly desirable for characterization of not-yet sequenced genomes like those of dinoflagellates. Using data from completely sequenced and annotated genomes from phylogenetically diverse lineages, we investigated the relationship between gene content and genome size using regression analyses. Distinct relationships between log_10_-transformed protein-coding gene number (Y′) versus log_10_-transformed genome size (X′, genome size in kbp) were found for eukaryotes and non-eukaryotes. Eukaryotes best fit a logarithmic model, Y′ = ln(-46.200+22.678X′, whereas non-eukaryotes a linear model, Y′ = 0.045+0.977X′, both with high significance (p<0.001, R^2^>0.91). Total gene number shows similar trends in both groups to their respective protein coding regressions. The distinct correlations reflect lower and decreasing gene-coding percentages as genome size increases in eukaryotes (82%–1%) compared to higher and relatively stable percentages in prokaryotes and viruses (97%–47%). The eukaryotic regression models project that the smallest dinoflagellate genome (3×10^6^ kbp) contains 38,188 protein-coding (40,086 total) genes and the largest (245×10^6^ kbp) 87,688 protein-coding (92,013 total) genes, corresponding to 1.8% and 0.05% gene-coding percentages. These estimates do not likely represent extraordinarily high functional diversity of the encoded proteome but rather highly redundant genomes as evidenced by high gene copy numbers documented for various dinoflagellate species.

## Introduction

An increasing amount of evidence supports a general positive correlation between gene content and genome size in prokaryotes and small eukaryotes, but whether this trend applies to all eukaryotes has been questioned and remains to be investigated [1]–[3]. As genome size can be measured easily, a robust correlation between gene content and genome size would provide a simple tool for predicting gene contents of not-yet sequenced genomes such as those of dinoflagellates. Dinoflagellates are one of the largest algal groups in the ocean, contributing significantly to oceanic primary production and coral reef building. Dinoflagellates are ecologically and economically important also because many of them form harmful algal blooms and even produce toxins. Among many unique characteristics, dinoflagellates possess unusually large genomes [4]. Although smaller genomes may occur in some yet unrecognized dinoflagellates [5], the typical dinoflagellate genomes are larger than most eukaryotes examined to date. The smallest documented dinoflagellate genomes are found in the coral reef symbiont *Symbiodinium* spp., ranging from 1.5 to 4.8 (average ∼3) pg DNA per haploid genome [6], while the largest (250 pg DNA per haploid genome) is found in *Prorocentrum micans* [7]. Equivalent to 3–245×10^6^ kbp per haploid genome, dinoflagellate genomes are about 1–77 fold that of the human haploid genome, and greater than any other algal groups (∼13–200×10^3^ kbp) by a factor of hundreds to thousands [6]–[10]. It has been suggested that the large fraction of the dinoflagellate genomes are nonfunctional repeated DNA sequences [9], [11]–[15]. How many genes are encoded in the genomes of these unicellular and seemingly simple organisms remains a question, which potentially bears significance on eukaryotic genome evolution. Information on gene contents of dinoflagellate genomes will allow researchers to gain understanding on how the large genomes favor or disfavor these organisms in their wide range of habitats.

Unfortunately, the infeasibility of sequencing these gigantic genomes with the current technology has hindered the progress in understanding dinoflagellate gene content. The next generation technologies such as 454, Solexa, or SOLiD™ are promising in reducing the enormous costs needed to sequence a dinoflagellate genome. However, the challenge in assembling the relatively short fragments is still insurmountable especially because in dinoflagellates many genes occur in numerous highly similar copies [16], [17]. Predictably, it will not be so soon before a dinoflagellate genome can be completely sequenced and accurately assembled to give a correct gene count. Any indirect approach to provide gene content estimate is desirable presently.

Taking advantage of the rapidly growing genome sequence dataset, we analyzed the relationship between gene content and genome size in all sequenced life forms. We then used the resultant eukaryotic regression equations to estimate gene content for dinoflagellate genomes. In light of high gene copy numbers reported for various dinoflagellates, implications of the high gene numbers and possible evolutionary mechanisms giving rise to the enormous genomes in this phylum is discussed.

## Methods

### Data collection

Data up to date by February 2009 were retrieved from the Reference Sequence (RefSeq) collection in the National Center for Biotechnology information (NCBI; http://www.ncbi.nlm.nih.gov), the Integrated Microbial Genomes (IMG) system in DOE Joint Genome Institute (JGI; http://img.jgi.doe.gov), and peer-reviewed publications (Supplemental Table S1). Dataset included total number of nucleotide base pairs (i.e. genome size), number of protein-coding genes, and total number of genes (including protein-coding, rRNA, and tRNA), gene-coding percentage (percent of DNA bases that codes for genes in a genome) for 55 completely sequenced eukaryotic genomes and 1055 non-eukaryotic genomes including prokaryotes (478 from bacteria and 60 from archaea), viruses (260), and organelles (231 from mitochondria and 26 from chloroplasts). For gene-coding percentage, only data published in peer-reviewed articles were used in the analysis as data from JGI included introns and other untranslated regions and significantly overestimated gene-coding percentage in large eukaryotic genomes (Supplemental Table S1). Incomplete or draft genome sequence data were excluded from this study to avoid potential errors.

### Regression analyses and dinoflagellate gene content prediction

The genome size and gene number datasets were subject to Shapiro-Wilk and Kolmogorov-Smirnov normality tests using SPSS 15. When normality was violated, data were logarithmic-transformed. Regression analyses for logarithmic-transformed protein-coding (or total) gene number (dependent variables) versus log genome size (independent variable) were conducted using linear, logarithmic, and power regression models in SPSS 15. The intention was to seek an overall correlation for all genomes, but if it failed, to seek separate correlations for separate groups of genomes (e.g. eukaryotes and others). The different regression models were compared based on significance level and R^2^, and the best-fit model was selected. The established regression models were then used to predict dinoflagellate gene number based on documented genome size data (3–245×10^6^ kbp). Dinoflagellate gene-coding percentages were estimated based on this formula: (total gene number x average gene length/genome size)×100%, where average gene length was approximated as 1.346 kbp, a value previously found highly conserved in eukaryots [18].

## Results

### Distinct correlations between genome size and gene content for eukaryotes and non-eukaryotes

**Figure 1:**
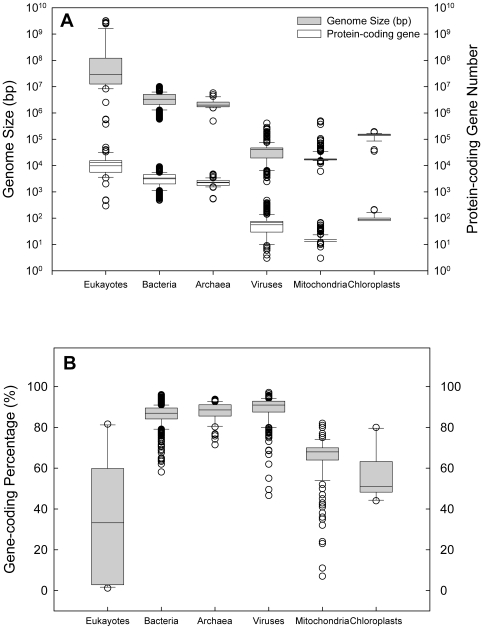
Genome sizes, protein-coding gene numbers, and gene-coding percentages of eukaryotic, bacterial, archaea, viral, and organellar genomes. (A) Genome size (shaded boxes) and number of protein-coding genes (open boxes). Total gene number is very close to protein-coding gene number and is not shown here. (B) Genome gene-coding percentage (fraction of DNA that constitutes genes). The lower and upper boundaries of the box indicate the first and third quartiles (or 25th and 75th percentiles) of each dataset, and the middle line in the box indicates the median value. The whiskers above and below the box indicate the 90th and 10th percentiles.

In the dataset we collected, the sequenced eukaryotic genomes ranged from 373 to 3,175,581 thousand base pairs (kbp) in size, while the genomes of non-eukaryotes (including bacteria, archaea, viruses, mitochondria, and chloroplasts) were substantially smaller, i.e., 2.4–9949.9 kbp (or kilobases in the case of single-stranded viral DNA or RNA) (Figure 1A). Correspondingly, total gene numbers were higher in eukaryotes than in non-eukaryotes (Figure 1A). The Shapiro-Wilk and Kolmogorov-Smirnov normality tests showed that the eukaryotic and non-eukaryotic genome sizes and total gene number were not of normal distribution. Thus, logarithmic-transformed data were used in further analysis.

**Figure 2:**
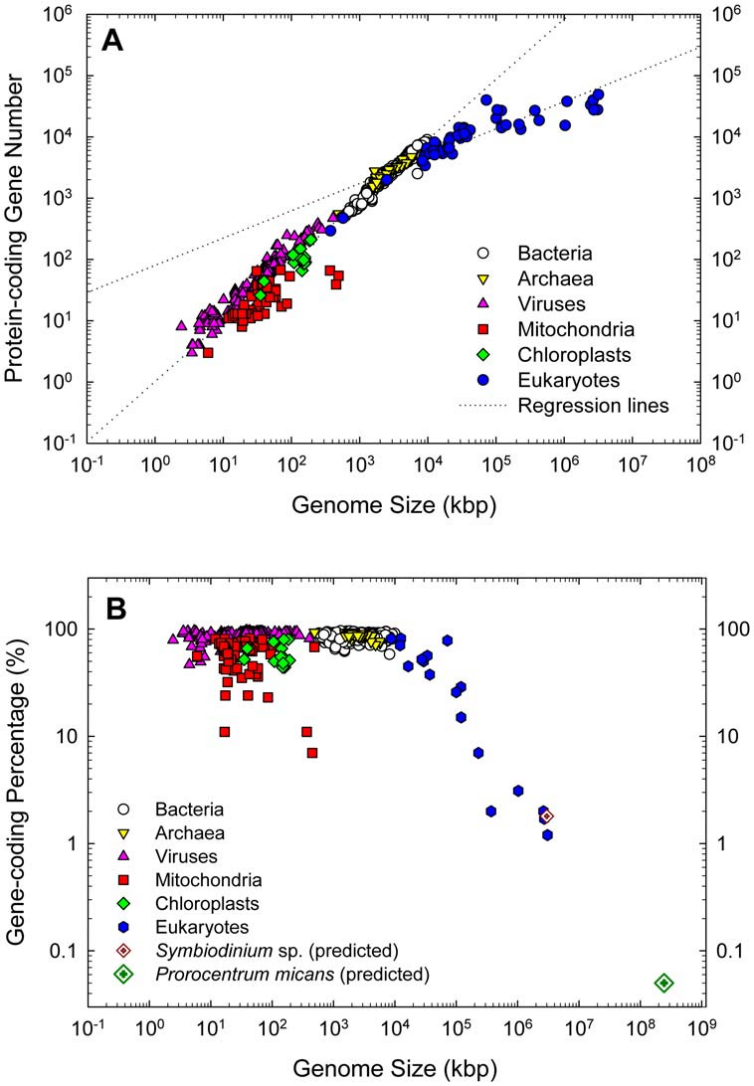
Distinct relationships between genome features in sequenced eukaryotes and non-eukaryotes. All correlations were highly significant (*p*<0.001). (A) Protein-coding gene number vs. genome size regression lines on log scale. Separate regression lines were yielded for eukaryotes (blue circles) and the non-eukaryotes (prokaryotes, viruses, and organelles; other symbols). (B) Gene-coding percentage vs. genome size on log scale. Note the negative trend for the eukaryotic genomes. The projected gene-coding percentage for the smallest (*Symbiodinium* sp., 1.80%) and largest dinoflagellate (*Prorocentrum micans*, 0.05%) genomes calculated based on reported average eukaryotic gene length (1.346 kbp) are shown for comparison. The trend for the non-eukaryotes is almost horizontal except for the outliers from some organelles.

**Figure 3:**
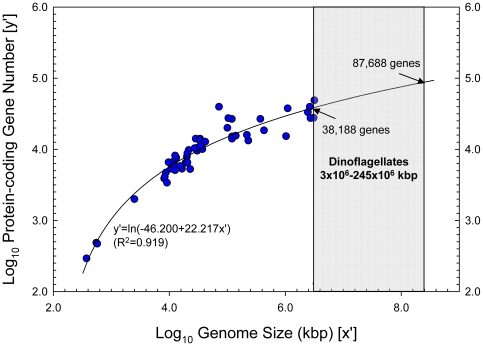
Logarithmic regression model for log_10_-transformed eukaryotic gene number (y′) versus log_10_-transformed genome size (x′). Range of dinoflagellate genome size (3×10^6^–245×10^6^ kbp) is indicated by the shaded areas. The predicted gene numbers for the recognized smallest (38,188) and largest (87,688) dinoflagellate genomes correspond to their gene-coding percentages shown in Fig. 2B.

When the log_10_-transformed data of gene number were plotted against log_10_ genome size, two distinct relations appeared: eukaryotes in one and non-eukaryotes in the other, with markedly different slopes emerging from initial linear regressions (Fig. 2A). Therefore, further multi-model analyses were performed separately for these two groups. For non-eukaryotes, the linear regression model was best fit (p<0.001, highest R^2^) among all the different models examined (Table 1). For eukaryotes, the log_10_-transformed data best fit a natural logarithmic (ln) regression model (Table 1, Figure 3). As the protein-coding gene number was generally very close to the total gene number in each genome, similar significant positive correlations were found for total gene numbers in both eukaryotic and non-eukaryotic genomes (Table 1), although only the protein-coding gene number is shown in the figures (Figure 2A, 3).

**Table 1:** Summary of regression models with best fit models for each group italicized.

Model^a^	Regression equation^b^	R^2^	Estimated gene	dinoflagellate numbers
			Smallest (3×10^6^ kbp)	Largest (245×10^6^ kbp)
**Eukaryotes**				
Protein coding genes (n = 55)				
linear	y′ = 1.902+0.445x′	0.795		
*logarithmic*	*y*′* = ln(−46.20+22.22x*′*)*	*0.919*	*38188*	*87688*
power	ln(y′) = 1.629+0.583ln(x′)	0.853		
Total genes (n = 48)				
linear	y′ = 1.802+0.470x′	0.857		
*logarithmic*	*y*′* = ln(−47.28+22.74x*′*)*	*0.924*	*40086*	*92013*
power	ln(y′) = 1.602+0.597ln(x′)	0.900		
**Non-eukaryotes**				
Protein coding genes (n = 1051)				
*linear*	*y*′* = 0.045+0.977x*′	*0.984*		
logarithmic	y′ = 0.840+2.051ln(x′)	0.954		
power	ln(y′) = 1.012+0.980ln(x′)	0.963		
Total genes (n = 1051)				
*linear*	*y*′* = 0.379+0.884x*′	*0.987*		
logarithmic	y′ = 1.096+1.855ln(x′)	0.958		
power	ln(y′) = 1.227+0.828ln(x′)	0.968		

a p<0.001 for all models; n = sample size.

b y′ = log_10_ gene number (y, protein coding or total gene number) and x′ = log_10_ genome size (x, in kbp).

On the contrary, the gene-coding fraction of the genome, i.e., gene-coding percentage, showed a different trend against genome size than the gene number trend (Figure 1B, 2B). In eukaryotes, the gene-coding percentage declined from 81.6% to 1.2% as the genome size increased (Figure 2B, Supplemental Table S1). The gene-coding percentage in non-eukaryotes was generally higher (97%–47%) and varied markedly less with genome size (Figure 1B, 2B) than in eukaryotes. The only exceptions were the organellar genomes, which exhibited a substantially lower gene-coding percentage than prokaryotes and viruses, indicating disproportionate loss of coding sequences during organellar genome reduction.

### Dinoflagellate gene content estimation

The high R^2^ and low p values (<0.001) in the log_10_ gene number versus log_10_ genome size regression models (Table 1) suggested that the empirically derived correlations were highly significant and could be used to make valid predictions of gene numbers. As the smallest recognized dinoflagellate genome (3×10^6^ kbp, in *Symbiodinium* spp.) falls within the range of genome sizes used to derive the eukaryotic correlation, the regression equation can be applied directly, which gave 38,188 protein-coding (40,086 total) genes per genome. For the largest documented dinoflagellate genome (245×10^6^ kbp, in *P. micans*), the empirical regression equation needed to be extrapolated with the assumption that the same correlation holds for larger genomes. As a result, the gene number estimate was 87,688 protein-coding (92,013 total) genes (Figure 3). Based on the previously reported average eukaryotic gene length, 1.346 kbp [18], these gene number estimates corresponded to 1.80% and 0.05% respectively for the smallest and the largest dinoflagellate genomes (Figure 2B).

## Discussion

### Distinction and robustness of regression models

Statistical analyses on up-to-date sequenced genome data show the lack of a universal correlation covering all life forms, in agreement with previous studies [1]–[3]. Our results further present evidence, for the first time, of an overall correlation in eukaryotic genomes between log_10_ gene number and log_10_ genome size. The best-fit regression model for log_10_-transformed eukaryote data is a log_e_ function and that for log_10_-transformed non-eukaryote data is a linear function, two distinct relationships. This indicates that as genome size increases the number of genes increases at a disproportionately slower rate in eukaryotes than in non-eukaryotes. In another word, the proportion of non-coding DNA increases with genome size faster in eukaryotes than in non-eukaryotes. This is consistent with the previous findings that the vast majority of nuclear DNA in eukaryotes is non-gene-coding elements including introns, pseudogenes, and transposable elements whereas prokaryotic, viral, and organellar genomes are mostly composed of gene-coding sequences [1], [3].

The smallest eukaryotic genomes collected in this study are from the nucleomorphs of *Bigelowiella natans* (373 kbp), *Guillardia theta* (551 kbp), and *Hemiselmis andersenii* (572 kbp) followed by the parasitic fungus *Encephalitozoon cuniculi* (2,500 kbp). Their gene numbers and genome sizes are comparable to some bacteria (Figure 2). The nucleomorph is a remnant nucleus of the secondary endosymbiont that has evolved to a chloroplast in the host crytophyte and chlorarachniophyte algae [19]. While the counterparts in other lineages of algae have been completely lost, nucleomorphs in these two lineages remain, but the sizes of their genomes have remarkably reduced. For *E. cuniculi*, its small genome may be a result of selection for a minimal genome size in parasitism evolution. Gene numbers of these small eukaryotic genomes appear to also fit on the non-eukaryotic regression lines (Fig. 2A), suggesting that nuclear genome reduction during chloroplast and parasitism evolution has resulted in elevated gene density. This is the reverse of genome expansion that results from disproportionate increase of non-gene-coding DNA [1], [3]. The two largest eukaryotic genomes analyzed were about 3,175,581 kbp in the primate *Pan troglodytes* and 3,080,436 kbp in humans, 8,514 times larger than the smallest (*B. natans* nucleomorph). Genome sequencing probably has biased toward relatively small genomes, as indicated by limited number of sequenced genomes larger than humans'; however, the current dataset cover a wide genome size, phylogenetic, and ecological ranges. The high statistical significance and R^2^ value of the log_10_ gene number- log_10_ genome size correlation derived from this dataset suggests that the resultant regression equation should provide reliable predictions on gene numbers for many species.

### Predicting power of the eukaryotic regression model for dinoflagellate genomes

A question about applying the eukaryotic regression model to dinoflagellate genomes stems from potential effects of distinct dinoflagellate genome organization on the log_10_ gene number-log_10_ genome size correlation. Unique among eukaryotes, dinoflagellate genomes have a few to over 200 chromosomes, which are permanently condensed, and not organized by nucleosomes [20]. The condensed chromosomes show a striating banding pattern under electron microscope that result from liquid cholesteric DNA crystal, which are formed by stacked disks of parallel bundles of DNA filaments that make a continuous left-handed twist along the chromosome's longitudinal axis [21]. Histone-like basic DNA-binding proteins are probably involved in stabilizing this structure by neutralizing local electronegative charges that would result from tightly compacted DNA filaments [22]. While most of this DNA is believed to be transcriptionally inactive, at the periphery of these disks are loops of DNA that are less tightly compacted and actively transcribed [23], [24]. As mentioned earlier, most of the dinoflagellate genes studied so far are organized in tandem repeats, not so commonly seen in eukaryotes. Dinoflagellate genomes also host complex molecular machinery of mRNA editing [25] and spliced leader (SL) *trans*-splicing [26 and ref therein].

While no information is available to prove whether these genomic features will lead to alteration of the log_10_ gene number - log_10_ genome size relationship, an examination on organisms sharing similar genomic features may provide some clue. Genomes of the kinetoplastids, which are phylogenetically distinct from dinoflagellates, share with dinoflagellates many of the unique genomic features, such as permanently condensed chromosomes, gene tandem repeat organizations, mRNA editing, and SL *trans*-splicing of transcripts [27]. Genomes of two kinetoplastid species, *Leishmania major* (32,800 kbp) and *Trypanosoma brucei* (26,000 kbp), have been sequenced, but data were not used in the regression analyses because the sequence annotation had not been finished at time of our data collection. The total gene numbers based on the draft genome sequences are 9,183 for *L. major* and 9,068 for *T. brucei* [28], [29], which are similar to what our eukaryotic regression model predicts (10,301 and 9,346, respectively). This comparison result indicates that the unique genome structures in this lineage will not cause significant deviation of genome features from the eukaryotic log_10_ gene number- log_10_ genome size relationship we have derived. It suggests that the relationship very likely holds for dinoflagellate genomes, particularly those of *Symbiodinium* spp. (∼3×10^6^ kbp), which are within the genome size range sampled in this study. The genomes of *Symbiodinium* spp. and some other modern dinoflagellates are shown to be haploid [30]–[35]. If polypoidy occurs in some dinoflagellates and accounts for their large nuclear genomes (see next section), practically gene contents in these species can also be estimated with their factored-down “haploid” genome sizes (if≤3×10^6^ kbp) using the regression equation developed here and the gene number estimate can then be factored up to the actual genome size. The equation can also be used to estimate the gene numbers for those having smaller genome size than *Symbidinium* spp. but yet to be identified [5].

Extrapolation of the regression model to accommodate genomes larger than sampled will have risk of overestimating or underestimating gene numbers, because the trend of the regression may possibly shift for large genomes like those of dinoflagellates. However, compared to a linear regression, the logarithmic regression we derived for eukaryotes inherently predicts a slower increase of gene number, and hence a progressively lower gene-coding percentage, as genome size increases. In fact, the predicted gene-coding percentages for the smallest and the largest dinoflagellate genome, 1.80% and 0.05% respectively, are remarkably lower than those for most other eukaryotes (1%–82%). Therefore, further leveling off of the regression line may not be so likely. A recent small-scale survey of *Heterocapsa triquetra* nuclear genome [36] is worth noting. Out of a 230 kbp sequence analyzed, 89.5% was non-repeated sequences with no similarity to any known genes but a 546-bp gene was identified. Applying the one per 230 kbp DNA gene density to the entire genome would yield about 91,500 genes for the 18.6–23.6×10^6^ (21.1×10^6^ on average) kbp *H. triquetra* nuclear genome. Alternatively, if we assume that the gene-coding percentage of this 230-kbp DNA (0.2%) and the previously reported eukaryotic average gene length (1.346 kbp) apply to this genome, the gene number would be 31,352. Our model-predicted 60,128 gene number for this species lies in the middle of the two extremes. Therefore, it seems unlikely that the eukaryote regression model we derived will seriously if at all overestimate gene numbers for large dinoflagellate genomes.

### Dinoflagellate gene contents and their implications in genome evolution

While all the available information point to a reasonable accuracy, or at least no overestimation, the model-predicted gene numbers for dinoflagellates (38,188–87,688 or about 1-3 fold as many as that in a human genome) are exceedingly high for these unicellular and therefore relatively “simple” organisms. However, these gene number estimates may not really represent an extraordinarily high functional diversity of the encoded proteome. A survey of literature reveals that previously examined dinoflagellate genes occur in 30–5,000 copies per genome (Table 2), indicating that high gene copy number is a widespread phenomenon in dinoflagellate genomes. The sequences of these gene copies may be identical in some cases like the rRNA locus but slightly different from each other in most cases. Regardless, the widespread gene duplicates may offset the high total protein-coding gene numbers, giving a reasonable number of unique genes compared to what is expected of a typical unicellular eukaryote.

**Table 2:** Dinoflagellate gene copy numbers documented to date.

Gene	Species	Copy number per genome**	Reference
Actin*	*Amphidinium carterae*	≥113	[49]
Protein kinase gene	*Lingulodinium polyedrum*	∼30	[50]
Form II Rubisco gene*	*Prorocentrum minimum*	148	[16]
Luciferase gene*	*Alexandrium affine*	60	[51]
	*Alexandrium tamarense*	126	[51]
	*Lingulodinium polyedrum*	146	[51]
	*Pyrocystis fusiformis*	44	[51]
	*Pyrocystis lunula*	160	[51]
	*Pyrocystis noctiluca*	110	[51]
	*Protoceratium reticulatum*	48	[51]
Luciferin-binding protein gene	*Lingulodinium polyedrum*	∼1,000	[52]
Mitotic cyclin gene	*Lingulodinium polyedrum*	∼5,000	[53]
Peridinin-chlorophyll *a* binding protein gene*	*Lingulodinium polyedrum*	∼5,000	[54]
	*Symbiodinium* sp. 203	36	[55]
Proliferating cell nuclear antigen gene*	*Pfiesteria piscicida*	41	[17]

*arranged in tandem repeats.

**A. carterae actin copy number was based on cloning and sequencing (Figure 4 in [49]); all other gene copy numbers here were based on probe hybridization or quantitative PCR.

While little genomic data are available to support this proposition, some insights can be obtained from EST data that have been generated for several dinoflagellate species. Typically in these studies EST sequences in each species were clustered at an identity cutoff around 95%, which is expected to group cDNA copies into unique (or semi-unique) transcripts. In *Alexandrium tamarense* (genome size 200×10^6^ kbp), 6,723 unique transcripts were identified out of a 11,171-EST dataset [37]; in *Heterocapsa triquetra* (about 20×10^6^ kbp), 2,022 unique clusters were assembled out of 6,765 sequenced ESTs [38]; in *Karenia brevis* (about 100×10^6^ kbp), 11,937 unique out of 25,000 ESTs [39]; in *K. veneficum* (formerly *K. micrum*; 5×10^6^ kbp), 11,903 unique out of 16,544 [40]; in *Oxyrrhis marina* (genome size unknown), 9,876 unique out of 18,012 [41]. True unique-gene numbers of these species likely are higher than these unique-transcript numbers because an EST dataset does not include genes not expressed at time of sampling, and furthermore, as the sequencing scales in these projects were relatively small the data likely only account for a fraction of the expressed gene pool missing those expressed at lower levels. Nevertheless, these incomplete EST data reveal a minimum of nearly 12,000 unique genes even for the relatively small dinoflagellate genome of *K. veneficium* (∼5×10^6^ kbp). In this case, if the average gene copy number is 3, the 42,770 protein-coding genes predicted by our regression model would represent a collection of 14,257 unique genes, a number close to the EST-based unique gene estimate (>12,000).

Many questions remain regarding dinoflagellate genome composition and its evolution. As the gene-coding percentage is very low, the large and widely ranged dinoflagellage genome sizes are clearly not due to the high gene numbers we predicted here. Non-coding DNA (e.g. repetitive sequences, introns, transposons) dominates the genomes as in any large eukaryote genomes, attested to by the abundant transposable elements found in a small fraction of *H. triquetra* genomic DNA [36]. On the contrary, the high gene numbers, especially high gene copy numbers, is likely the result of genome expansion. It is believed that dinoflagellate genomes have been subject to duplications of individual genes or segmental to whole genome duplication [5], [39], or combinations of these mechanisms. Tandem-repeated genes, like those that have been studied in dinoflagellates (Table 2), are more likely to have resulted from successive gene duplications through unequal cross-over of chromosomes [16]. In addition, it is possible that dinoflagellate genomes can take up and incorporate cDNAs, resulting in multiplication of genes such as that coding for SL [42]. However, location of gene copies on separate chromosomes is evident at least in the case of Rubisco in *Prorocentrum minimum*, suggesting possible duplication at chromosomal level or higher [16]. Whole genome duplications by autopolyploidy or allopolyploidy events are the most efficient mechanism to introduce extra genetic material and significantly expand the genomes [43], and have been well documented for animals, plants and protists such as the budding yeast *Saccharomyces cerevisiae* and the ciliate *Paramecium tetraurelia* [44]–[47]. Given the widespread gene repetition in dinoflagellates, genome duplication is very possible. In fact, ancient polyploidy has been suggested as a mechanism of speciation in the dinoflagellate *Heterocapsa pygmaea* [48]. Because usually most gene duplicates are eventually lost or diverged to different genes after genome duplication, the retention of the numerous copies of genes in dinoflagellates may indicate an evolutionary driving force associated with functional requirements imposed on dinoflagellates for adaptation to a wide range of habitats. In support of this, highly expressed genes tend to occur in tandem-repeated copies [16], [49]. The predicted high gene numbers can be a result of gene and genome duplication followed by differential gene loss and diversification. Ultimate verification of actual gene number and genome duplication as a potential causative mechanism would require sequencing of one or more dinoflagellate genomes, which will also further validate the eukaryotic log gene number-log genome size correlation empirically derived in this study.

## Supporting Information

Table S1 Genome size, protein-coding gene number, total gene number, and gene-coding percentage for the sequenced genomes of eukaryotes, bacteria, archaea, viruses, mitochondria, and chloroplasts estimated based on genome sequences.
(1.97 MB DOC)
