# Supplementary material for: Distinct Gene Number-Genome Size Relationships for Eukaryotes and Non-Eukaryotes: Gene Content Estimation for Dinoflagellate Genomes
Source: PLoS One. 2009 Sep 14;4(9):e6978. doi: 10.1371/journal.pone.0006978 (PMC2737104; doi:10.1371/journal.pone.0006978)
Supplement: Table S1 — Genome size, protein-coding gene number, total gene number, and gene-coding percentage for the sequenced genomes of eukaryotes, bacteria, archaea, viruses, mitochondria, and chloroplasts estimated based on genome sequences. (1.97 MB DOC) [file pone.0006978.s001.doc]

Supplemental Table **S1**. Genome size, protein-coding gene number, total gene number, and gene-coding percentage for the sequenced genomes of eukaryotes, bacteria, archaea, viruses, mitochondria, and chloroplasts estimated based on genome sequences.

| **Species or strain** | **Genome size (kbp)** | **Protein-coding gene number** | **Total gene number*** | **Gene-coding percentage**** |
| --- | --- | --- | --- | --- |
| **Eukaryotes** |  |  |  |  |
| *Anopheles gambiae PEST* | 230466.9 | 13260 | 13749 | 19.11 (7.0) |
| *Apis mellifera* DH4 | 217194.9 | 15948 | 16360 |  |
| *Arabidopsis thaliana* columbia | 119707.4 | 26735 | 31392 | 49.99 (28.8) |
| *Aspergillus fumigatus* Af293 | 29384.9 | 9923 | 10152 | 55.39 (50.1) |
| *Aspergillus nidulans* FGSC A4 | 30166.8 | 9541 | 9727 | 59.16 |
| *Aspergillus niger* CBS 513.88 | 33975.5 | 14086 | 14431 | 65.46 |
| *Aspergillus terreus* NIH2624 | 29164.9 | 10406 | 10556 | 59.92 |
| *Bigelowiella natans* nucleomorph | 372.9 | 293 | 340 |  |
| *Bombyx mori* | 428700 | 18510 |  |  |
| *Caenorhabditis elegans* | 100281.2 | 20056 | 20935 | 59.88 (25.8) |
| *Candida albicans* SC5314 | 28504.7 | 14105 | 14144 | 63.97 |
| *Candida glabrata* CBS 138 | 12280.4 | 5181 | 5397 | 64.37 |
| *Canis familiaris* | 2445110.1 | 33202 | 50110 |  |
| *Chlamydomonas reinhardtii* | 121000 | 15143 |  |  |
| *Cryptococcus neoformans* neoformans JEC21 | 19051.9 | 6475 | 6609 | 66.88 |
| *Cryptococcus neoformans* var. neoformans B-3501A | 19699.8 | 6500 | 6609 | 64.93 |
| *Cryptosporidium hominis* TU502 | 8171.0 | 3886 | 3956 | 64.91 |
| *Cryptosporidium parvum* Iowa type II | 9098.4 | 3396 | 3886 | 68.90 |
| *Cyanidioschyzon merolae* 10D | 16520.3 | 5331 | 5373 | (44.90) |
| *Danio rerio* Tuebingen | 1097524.4 | 37724 | 38017 | 38.23 |
| *Debaryomyces hansenii* CBS767 | 12220.8 | 6893 | 7108 | 76.14 |
| *Dictyostelium discoideum* AX4 | 33928.5 | 13322 | 13362 | 68.76 (56.3) |
| *Drosophila melanogaster* | 120401.1 | 14081 | 21116 | 61.37 (15.0) |
| *Encephalitozoon cuniculi* GB-M1 | 2497.5 | 1996 | 2053 | 86.99 |
| *Entamoeba histolytica* HM-1:IMSS | 21581.5 | 9772 | 10202 | 54.53 |
| *Eremothecium gossypii* | 8742.4 | 4718 | 4987 | 80.17 (81.2) |
| *Gallus gallus* | 1031880.1 | 15288 | 15700 | (3.1) |
| *Giardia lamblia* ATCC 50803 | 9717.5 | 6569 | 6598 | 94.45 |
| *Gibberella zeae* PH-1 | 36487.3 | 11640 | 11640 | 55.64 |
| *Guillardia theta* nucleomorph | 551.3 | 486 | 553 | 90.34 |
| *Hemiselmis andersenii* CCMP644 nucleomorph | 572 | 472 | 525 |  |
| *Homo sapiens* | 3080436 | 27727 | 38612 | 35.34 (1.20) |
| *Kluyveromyces lactis* NRRL Y-1140 | 10689.2 | 5331 | 5502 | 70.93 |
| *Magnaporthe grisea* 70-15 | 41495.9 | 12832 | 13146 | 47.88 |
| *Mus musculus* C57BL/6J | 2644094.0 | 39625 | 60745 | 35.34 (2.0) |
| *Neurospora crassa* OR74A | 37097.3 | 10079 | 10082 | 45.49 (37.6) |
| *Oryza sativa* (japonica cultivar-group) | 370792 | 26777 | 28431 |  |
| *Ostreococcus lucimarinus* CCE9901 | 13204.9 | 7603 | 7640 | 75.31 |
| *Ostreococcus tauri* | 12560 | 8166 |  | (81.60) |
| *Pan troglodytes* | 3175580.7 | 48911 | 49446 |  |
| *Paramecium tetraurelia* (micronuclear) | 72000 | 39642 |  | (78) |
| *Phaeodactylum tricornutum* CCAP1055/1 | 27400 | 10402 |  | (52) |
| *Pichia stipitis* CBS 6054 | 15441.2 | 5816 | 5816 | 61.23 |
| *Plasmodium falciparum* 3D7 | 22859.5 | 5268 | 5298 | 58.38 |
| *Plasmodium yoelii* yoelii 17XNL | 20087.0 | 7860 | 7910 | 59.12 |
| *Rattus norvegicus* GK/Ox | 2718897.3 | 27490 | 38115 | 31.44 (1.7) |
| *Saccharomyces cerevisiae* | 12156.6 | 5869 | 6276 | 73.57 (70.5) |
| *Schizosaccharomyces pombe* 972h- | 12554.3 | 5093 | 5364 | 60.42 |
| *Tetrahymena thermophila* (macronuclear) | 104100 | 27424 | 28345 |  |
| *Thalassiosira pseudonana* CCMP1335 | 34500 | 11242 |  |  |
| *Theileria parva* Muguga | 8347.6 | 4079 | 4159 | 82.14 |
| *Trypanosoma brucei* TREU927 | 20527.4 | 8772 | 10253 | 51.03 |
| *Ustilago maydis* 521 | 19638.0 | 6522 | 6631 | 64.33 |
| *Volvox carteri* | 140000 | 15544 |  |  |
| *Yarrowia lipolytica* CLIB122 | 20503.0 | 6520 | 7042 | 47.15 |
|  |  |  |  |  |
| **Bacteria** |  |  |  |  |
| *Acidiphilium cryptum* JF-5 | 3917.1 | 3551 | 3552 | 89.55 |
| *Acidothermus cellulolyticus* 11B | 2445.4 | 2151 | 2197 | 90.39 |
| *Acinetobacter baumannii* ATCC 17978 (chr) | 3976.7 | 3352 | 3439 | 71.00 |
| *Acinetobacter* sp. ADP1 | 3598.6 | 3325 | 3422 | 88.85 |
| *Actinobacillus pleuropneumoniae* 4074 | 2292.3 | 2132 | 2193 | 86.03 |
| *Actinobacillus pleuropneumoniae* L20 | 2274.5 | 2012 | 2108 | 86.00 |
| *Actinobacillus succinogenes* 130Z | 2046.1 | 1884 | 1938 | 89.32 |
| *Agrobacterium tumefaciens* C58 Cereon | 5673.5 | 5301 | 5366 | 89.60 |
| *Agrobacterium tumefaciens* C58 Dupont | 5674.1 | 5402 | 5467 | 88.45 |
| *Alkalilimnicola ehrlichei* MLHE-1 | 3272.8 | 2869 | 2923 | 90.66 |
| *Alkaliphilus metalliredigenes* QYMF | 4410.3 | 4090 | 4233 | 82.44 |
| *Anabaena* PCC7120 | 7211.8 | 6132 | 6214 | 82.41 |
| *Anabaena variabilis* ATCC29413 | 7105.8 | 5746 | 5805 | 82.51 |
| *Anaeromyxobacter dehalogenans* 2CP-C | 5013.5 | 4361 | 4416 | 91.45 |
| *Anaplasma marginale* St. Maries | 1197.7 | 949 | 1005 | 85.88 |
| *Anaplasma phagocytophilum* HZ | 1471.3 | 1264 | 1304 | 68.62 |
| *Aquifex aeolicus* VF5 | 1590.8 | 1553 | 1603 | 92.68 |
| *Arthrobacter* sp. FB24 | 5011.6 | 4524 | 4591 | 90.16 |
| *Aster yellows* witches-broom phytoplasma AYWB | 724.0 | 693 | 728 | 74.81 |
| *Azoarcus* sp. EbN1 | 4727.3 | 4598 | 4668 | 90.89 |
| *Azotobacter vinelandii* AvOP | 5352.4 | 4990 | 5058 | 88.74 |
| *Bacillus anthracis* A2012 | 5093.6 | 5354 | 5390 | 81.08 |
| *Bacillus anthracis* Ames | 5227.3 | 5311 | 5439 | 81.71 |
| *Bacillus anthracis* Ames 0581 | 5503.9 | 5617 | 5745 | 81.13 |
| *Bacillus anthracis* Sterne | 5228.7 | 5287 | 5415 | 84.98 |
| *Bacillus cereus* ATCC 10987 | 5432.7 | 5844 | 6008 | 85.82 |
| *Bacillus cereus* ATCC 14579 | 5427.1 | 5255 | 5402 | 81.91 |
| *Bacillus cereus* E33L | 5300.9 | 5134 | 5269 | 85.14 |
| *Bacillus cereus* G9241 | 5934.9 | 6147 | 6316 | 81.24 |
| *Bacillus cereus* NVH391-98 | 3915.8 | 3886 | 4033 | 81.99 |
| *Bacillus clausii* KSM-K16 | 4303.9 | 4108 | 4205 | 86.95 |
| *Bacillus halodurans* C-125 | 4202.4 | 4066 | 4172 | 86.05 |
| *Bacillus licheniformis* Goettingen | 4222.6 | 4196 | 4289 | 87.89 |
| *Bacillus licheniformis* Novozymes | 4222.3 | 4152 | 4245 | 86.78 |
| *Bacillus subtilis* 168 | 4214.6 | 4106 | 4225 | 88.30 |
| *Bacillus thuringiensis* konkukian 97-27 | 5237.7 | 5117 | 5264 | 85.15 |
| *Bacillus weihenstephanensis* KBAB4 | 5602.5 | 5532 | 5629 | 81.97 |
| *Bacteroides fragilis* NCTC9343 | 5241.7 | 4308 | 4400 | 89.74 |
| *Bacteroides fragilis* YCH46 | 5311.0 | 4625 | 4717 | 90.52 |
| *Bacteroides thetaiotaomicron* VPI-5482 | 6293.4 | 4816 | 4902 | 89.88 |
| *Bartonella henselae* Houston-1 | 1931.0 | 1612 | 1664 | 76.73 |
| *Bartonella quintana* Toulouse | 1581.4 | 1308 | 1357 | 78.72 |
| *Bdellovibrio bacteriovorus* HD100 | 3783.0 | 3583 | 3625 | 93.14 |
| *Bifidobacterium longum* DJO10A | 2375.3 | 1956 | 2020 | 83.21 |
| *Bifidobacterium longum* NCC2705 | 2260.3 | 1729 | 1802 | 86.43 |
| *Blochmannia floridanus* | 705.6 | 589 | 635 | 84.91 |
| *Blochmannia pennsylvanicus* BPEN | 791.7 | 610 | 692 | 77.62 |
| *Bordetella bronchiseptica* RB50 | 5339.2 | 5006 | 5070 | 92.34 |
| *Bordetella parapertussis* 12822 | 4773.6 | 4402 | 4465 | 91.48 |
| *Bordetella pertussis* Tohama I | 4086.2 | 3806 | 3866 | 90.53 |
| *Borrelia burgdorferi* B31 | 1519.9 | 1639 | 1677 | 86.04 |
| *Borrelia garinii* Pbi | 986.9 | 932 | 972 | 92.29 |
| *Bradyrhizobium japonicum* USDA 110 | 9105.8 | 8317 | 8371 | 86.76 |
| *Bradyrhizobium* sp. BTAi1 | 8422.4 | 7462 | 7518 | 86.45 |
| *Brevibacterium linens* BL2 | 4510.7 | 3982 | 4038 | 86.42 |
| *Brucella abortus* 2308 | 3278.3 | 3350 | 3414 | 87.68 |
| *Brucella abortus* 9-941 | 3286.4 | 3085 | 3149 | 81.82 |
| *Brucella melitensis* 16M | 3294.9 | 3198 | 3264 | 86.67 |
| *Brucella suis* 1330 | 3315.2 | 3273 | 3337 | 84.98 |
| *Buchnera aphidicola* APS | 655.7 | 574 | 609 | 87.40 |
| *Buchnera aphidicola* Bp | 618.4 | 507 | 542 | 82.28 |
| *Buchnera aphidicola* Sg | 654.2 | 555 | 590 | 84.14 |
| *Burkholderia ambifaria* AMMD | 7503.6 | 6539 | 6617 | 88.28 |
| *Burkholderia cenocepacia* AU 1054 | 7249.5 | 6475 | 6555 | 88.10 |
| *Burkholderia cenocepacia* HI2424 | 8139.1 | 7065 | 7146 | 84.74 |
| *Burkholderia mallei* 10229 | 6065.2 | 5133 | 5201 | 79.68 |
| *Burkholderia mallei* 10399 | 5933.9 | 4886 | 4947 | 80.60 |
| *Burkholderia mallei* ATCC 23344 | 5835.5 | 4764 | 4831 | 77.88 |
| *Burkholderia mallei* FMH | 2273.4 | 2667 | 2687 | 78.90 |
| *Burkholderia mallei* GB8 horse 4 | 5804.9 | 4822 | 4884 | 80.48 |
| *Burkholderia mallei* JHU | 2190.5 | 2581 | 2608 | 79.81 |
| *Burkholderia mallei* NCTC 10247 | 5976.7 | 5096 | 5146 | 79.81 |
| *Burkholderia mallei* NCTC 10247 (chr1+2) | 5848.4 | 5852 | 5965 | 86.40 |
| *Burkholderia mallei* SAVP1 | 5283.5 | 4364 | 4438 | 80.98 |
| *Burkholderia pseudomallei* 1106a (chr1+2) | 7089.2 | 7183 | 7263 | 87.44 |
| *Burkholderia pseudomallei* 1655 | 7029.5 | 5410 | 5465 | 77.42 |
| *Burkholderia pseudomallei* 1710a | 7319.5 | 5502 | 5565 | 76.60 |
| *Burkholderia pseudomallei* 1710b | 7390.5 | 5479 | 5553 | 76.79 |
| *Burkholderia pseudomallei* 668 | 7071.9 | 5433 | 5495 | 78.59 |
| *Burkholderia pseudomallei* 668 (chr1+2) | 7040.4 | 7230 | 7307 | 87.89 |
| *Burkholderia pseudomallei* K96243 | 7247.5 | 5855 | 5928 | 83.62 |
| *Burkholderia pseudomallei* Pasteur | 7346.6 | 5595 | 5653 | 77.26 |
| *Burkholderia pseudomallei* S13 | 7388.3 | 5715 | 5771 | 78.01 |
| *Burkholderia* sp. 383 | 8676.3 | 7739 | 7824 | 87.74 |
| *Burkholderia thailandensis* E264 | 6724.0 | 5634 | 5704 | 86.95 |
| *Burkholderia vietnamiensis* G4 | 8411.0 | 7920 | 7992 | 87.75 |
| *Burkholderia xenovorans* LB400 | 9703.7 | 8930 | 9012 | 87.57 |
| *Burkholderia xenovorans* LB400 | 9731.1 | 8957 | 9039 | 87.52 |
| *Caldicellulosiruptor saccharolyticus* DSM 8903 | 2788.3 | 2602 | 2647 | 86.67 |
| *Campylobacter coli* RM2228 | 1860.7 | 1967 | 2019 | 92.25 |
| *Campylobacter jejuni* NCTC 11168 | 1641.5 | 1654 | 1707 | 95.41 |
| *Campylobacter jejuni* RM1221 | 1777.8 | 1838 | 1891 | 91.83 |
| *Campylobacter lari* RM2100 | 1562.9 | 1599 | 1651 | 94.54 |
| *Campylobacter upsaliensis* RM3195 | 1773.8 | 1934 | 1984 | 93.46 |
| *Carboxydothermus hydrogenoformans* Z-2901 | 2401.5 | 2620 | 2682 | 91.00 |
| *Caulobacter crescentus* CB15 | 4016.9 | 3737 | 3794 | 90.56 |
| *Chlamydia muridarum* Nigg | 1080.5 | 911 | 954 | 90.58 |
| *Chlamydia trachomatis* A/HAR-13 | 1052.0 | 919 | 960 | 90.96 |
| *Chlamydia trachomatis* D/UW-3/CX | 1042.5 | 894 | 937 | 90.83 |
| *Chlamydophila abortus* S26/3 | 1144.4 | 961 | 1002 | 90.98 |
| *Chlamydophila caviae* GPIC | 1181.4 | 1005 | 1047 | 89.78 |
| *Chlamydophila felis* Fe/C-56 | 1173.8 | 1013 | 1054 | 91.53 |
| *Chlamydophila pneumoniae* AR39 | 1229.9 | 1110 | 1151 | 89.22 |
| *Chlamydophila pneumoniae* CWL029 | 1230.2 | 1052 | 1118 | 88.76 |
| *Chlamydophila pneumoniae* J138 | 1226.6 | 1069 | 1134 | 90.05 |
| *Chlamydophila pneumoniae* TW-183 | 1225.9 | 1113 | 1154 | 90.55 |
| *Chlorobium limicola* DSMZ 245 | 2761.9 | 2436 | 2483 | 85.87 |
| *Chlorobium phaeobacteroides* BS1 | 4444.2 | 3791 | 3845 | 78.11 |
| *Chlorobium phaeobacteroides* DSMZ 266 | 3114.3 | 2792 | 2840 | 85.18 |
| *Chlorobium tepidum* TLS | 2154.9 | 2252 | 2308 | 88.46 |
| *Chlorobium vibrioforme* f. thiosulfatophilum DSMZ 265 | 1980.2 | 1747 | 1798 | 90.47 |
| *Chlorochromatium aggregatum* | 2572.1 | 2033 | 2081 | 89.07 |
| *Chloroflexus aurantiacus* J-10-fl | 5193.8 | 3938 | 3994 | 82.25 |
| *Chromobacterium violaceum* ATCC 12472 | 4751.1 | 4407 | 4530 | 89.40 |
| *Chromohalobacter salexigens* DSM 3043 | 3696.6 | 3319 | 3404 | 90.64 |
| *Chromohalobacter salexigens* DSM 3043 | 3671.0 | 3347 | 3422 | 90.92 |
| *Clostridium acetobutylicum* ATCC 824 | 4132.9 | 3848 | 3955 | 86.93 |
| *Clostridium beijerincki* NCIMB 8052 | 5952.5 | 5068 | 5210 | 80.61 |
| *Clostridium difficile* 630 | 4290.3 | 3742 | 3971 | 82.00 |
| *Clostridium perfringens* 13 | 3085.7 | 2723 | 2849 | 85.10 |
| *Clostridium tetani* E88 | 2873.3 | 2432 | 2504 | 85.96 |
| *Clostridium thermocellum* ATCC 27405 | 3895.0 | 3163 | 3235 | 79.84 |
| *Clostridium thermocellum* ATCC 27405 | 3843.3 | 3191 | 3307 | 83.00 |
| *Colwellia psychrerythraea* 34H | 5373.2 | 4910 | 5114 | 85.36 |
| *Corynebacterium diphtheriae* NCTC 13129 | 2488.6 | 2320 | 2389 | 90.30 |
| *Corynebacterium efficiens* YS-314 | 3219.5 | 2998 | 3069 | 90.83 |
| *Corynebacterium glutamicum* ATCC 13032 Kalinowski | 3282.7 | 3058 | 3136 | 88.37 |
| *Corynebacterium glutamicum* ATCC 13032 Nakagawa | 3309.4 | 3099 | 3177 | 87.83 |
| *Corynebacterium jeikeium* K411 | 2476.8 | 2120 | 2229 | 89.94 |
| *Coxiella burnetii* RSA 493 | 2032.7 | 2046 | 2091 | 84.29 |
| *Croceibacter atlanticus* HTCC2559 | 2954.7 | 2719 | 2756 | 92.63 |
| *Crocosphaera watsonii* WH8501 | 6285.4 | 5967 | 6011 | 78.22 |
| *Cupriavidus metallidurans* CH34 | 6887.6 | 6312 | 6377 | 88.89 |
| *Cupriavidus necator* (Ralstonia eutropha) JMP134 | 7255.3 | 6529 | 6613 | 88.53 |
| *Cytophaga hutchinsonii* ATCC 33406 | 4433.2 | 3592 | 3640 | 89.65 |
| *Dechloromonas aromatica* RCB | 4501.1 | 4204 | 4280 | 92.39 |
| *Dehalococcoides ethenogenes* 195 | 1469.7 | 1580 | 1629 | 90.20 |
| *Dehalococcoides* sp. BAV1 | 1299.5 | 1314 | 1354 | 89.44 |
| *Dehalococcoides* sp. CBDB1 | 1395.5 | 1458 | 1508 | 90.47 |
| *Deinococcus geothermalis* DSM11300 | 3164.1 | 2993 | 3058 | 89.53 |
| *Deinococcus radiodurans* R1 | 3284.2 | 3102 | 3159 | 87.71 |
| *Desulfitobacterium hafniense* DCB-2 | 6083.8 | 4391 | 4463 | 69.23 |
| *Desulfitobacterium hafniense* Y51 | 5727.5 | 5060 | 5137 | 85.21 |
| *Desulfotalea psychrophila* LSv54 | 3659.6 | 3236 | 3343 | 86.02 |
| *Desulfotomaculum reducens* MI-1 | 3467.3 | 3220 | 3272 | 83.57 |
| *Desulfovibrio desulfuricans* G20 | 3730.2 | 3784 | 3862 | 91.13 |
| *Desulfovibrio vulgaris* Hildenborough | 3773.2 | 3531 | 3615 | 86.57 |
| *Desulfuromonas acetoxidans* DSM 684 | 3529.0 | 3032 | 3065 | 87.31 |
| *Ehrlichia canis* Jake | 1315.0 | 944 | 983 | 72.91 |
| *Ehrlichia chaffeensis* Arkansas | 1176.2 | 1105 | 1145 | 79.94 |
| *Ehrlichia chaffeensis* sapulpa | 1005.8 | 805 | 834 | 72.99 |
| *Ehrlichia ruminantium* Gardel | 1499.9 | 980 | 1019 | 64.57 |
| *Ehrlichia ruminantium* str. Welgevonden | 1516.0 | 888 | 961 | 62.00 |
| *Ehrlichia ruminantium* Welgevonden ARC-OVI | 1516.4 | 943 | 982 | 63.32 |
| *Ehrlichia ruminantium* Welgevonden CIRAD | 1513.0 | 976 | 1015 | 63.94 |
| *Enterococcus faecalis* V583 | 3360.0 | 3264 | 3344 | 86.53 |
| *Enterococcus faecium* DO | 2848.4 | 2722 | 2794 | 84.40 |
| *Erwinia carotovora* subsp. atroseptica SCRI1043 | 5064.0 | 4492 | 4590 | 87.15 |
| *Erythrobacter litoralis* HTCC2594 | 3052.8 | 3042 | 3088 | 92.05 |
| *Escherichia coli* K12 | 4738.8 | 4359 | 4468 | 88.53 |
| *Escherichia coli* O157:H7 EDL933 | 5620.5 | 5449 | 5571 | 87.81 |
| *Escherichia coli* O6 CFT073 | 5231.4 | 5379 | 5489 | 88.66 |
| *Escherichia coli* Sakai O157:H7 | 5591.2 | 5444 | 5571 | 88.19 |
| *Escherichia coli* UTI89 | 5180.0 | 5211 | 5321 | 89.64 |
| *Exiguobacterium* sp. 255-15 | 2894.1 | 2976 | 3073 | 89.35 |
| *Flavobacterium johnsoniae* UW101 | 6069.8 | 4985 | 5037 | 87.26 |
| *Francisella tularensis* SCHU S4 | 1892.8 | 1804 | 1863 | 88.25 |
| *Frankia* sp. CcI3 | 5381.4 | 4561 | 4611 | 85.50 |
| *Frankia* sp. CcI3 | 5433.6 | 4548 | 4600 | 85.56 |
| *Frankia* sp. EAN1pec | 9081.4 | 7981 | 8039 | 85.68 |
| *Fusobacterium nucleatum* nucleatum ATCC 25586 | 2174.5 | 2067 | 2129 | 90.05 |
| *Fusobacterium nucleatum* subsp. vincentii ATCC 49256 | 2118.3 | 2250 | 2327 | 86.04 |
| *Geobacillus kaustophilus* HTA426 | 3592.7 | 3540 | 3654 | 86.10 |
| *Geobacter metallireducens* GS-15 | 4011.2 | 3576 | 3631 | 91.55 |
| *Geobacter metallireducens* GS-15 | 4011.2 | 3576 | 3631 | 91.55 |
| *Geobacter sulfurreducens* PCA | 3814.1 | 3447 | 3502 | 89.92 |
| *Geobacter uraniumreducens* Rf4 | 4882.3 | 4187 | 4188 | 85.94 |
| *Gloeobacter violaceus* PCC 7421 | 4659.0 | 4430 | 4478 | 89.31 |
| *Gluconobacter oxydans* 621H | 2922.4 | 2664 | 2732 | 90.02 |
| *Haemophilus ducreyi* 35000HP | 1699.0 | 1717 | 1784 | 86.62 |
| *Haemophilus influenzae* 86-028NP | 1913.4 | 1792 | 1869 | 88.53 |
| *Haemophilus influenzae* R2846 | 1824.2 | 1691 | 1759 | 89.11 |
| *Haemophilus influenzae* R2866 | 1933.3 | 1817 | 1892 | 89.24 |
| *Haemophilus influenzae* Rd KW20 | 1830.1 | 1709 | 1785 | 87.24 |
| *Haemophilus somnus* 129PT | 2008.4 | 1779 | 1845 | 89.59 |
| *Haemophilus somnus* 2336 | 2247.7 | 2081 | 2131 | 87.99 |
| *Hahella chejuensis* KCTC 2396 | 7215.3 | 6778 | 6860 | 88.20 |
| *Halorhodospira halophila* SL1 | 2674.3 | 2423 | 2423 | 92.10 |
| *Halothermothrix orenii* H 168 | 2464.0 | 2273 | 2274 | 87.08 |
| *Helicobacter hepaticus* ATCC 51449 | 1799.1 | 1875 | 1939 | 93.44 |
| *Helicobacter pylori* 26695 | 1667.9 | 1566 | 1609 | 90.04 |
| *Helicobacter pylori* J99 | 1643.8 | 1491 | 1531 | 90.69 |
| *Herminiimonas arsenicoxydans* | 3424.3 | 3325 | 3391 | 87.00 |
| *Idiomarina loihiensis* L2TR | 2839.3 | 2628 | 2697 | 92.80 |
| *Jannaschia* sp. CCS1 | 4404.0 | 4283 | 4328 | 90.77 |
| *Jannaschia* sp.CCS1 | 4399.7 | 4292 | 4337 | 90.87 |
| *Kineococcus radiotolerans* SRS30216 | 4894.0 | 4561 | 4618 | 90.69 |
| *Lactobacillus acidophilus* NCFM | 1993.6 | 1864 | 1944 | 89.27 |
| *Lactobacillus brevis* ATCC367 | 1880.8 | 1550 | 1600 | 72.96 |
| *Lactobacillus casei* ATCC334 | 2760.7 | 2478 | 2529 | 71.74 |
| *Lactobacillus delbrueckii* bulgaricus ATCCBAA-365 | 1629.4 | 1543 | 1622 | 73.33 |
| *Lactobacillus gasseri* ATCC33323 | 1950.2 | 1695 | 1757 | 84.09 |
| *Lactobacillus johnsonii* NCC 533 | 1992.7 | 1820 | 1915 | 90.85 |
| *Lactobacillus plantarum* WCFS1 | 3308.3 | 3051 | 3137 | 85.25 |
| *Lactobacillus reuteri* 100-23 | 2174.3 | 1972 | 2037 | 85.95 |
| *Lactobacillus reuteri* JCM 1112 | 1788.9 | 1707 | 1758 | 85.97 |
| *Lactobacillus reuteri* JCM 1112 | 1788.9 | 1707 | 1758 | 85.97 |
| *Lactobacillus sakei* sakei 23K | 1884.7 | 1884 | 1968 | 88.73 |
| *Lactobacillus salivarius* UCC118 | 2134.0 | 2017 | 2116 | 84.39 |
| *Lactococcus lactis* cremoris SK11 | 2613.2 | 2628 | 2690 | 73.03 |
| *Lactococcus lactis* lactis Il1403 | 2365.6 | 2266 | 2346 | 85.88 |
| *Legionella pneumophila* Lens | 3345.7 | 2947 | 2999 | 88.58 |
| *Legionella pneumophila* Paris | 3503.6 | 3082 | 3134 | 88.41 |
| *Legionella pneumophila* Philadelphia 1 | 3397.8 | 2942 | 2997 | 88.67 |
| *Leifsonia xyli* xyli CTCB07 | 2584.2 | 2030 | 2078 | 70.42 |
| *Leptospira interrogans* serovar Copenhageni Fiocruz | 4627.4 | 3658 | 3700 | 74.98 |
| *Leptospira interrogans* serovar Lai 56601 | 4691.2 | 4725 | 4766 | 78.18 |
| *Leuconostoc mesenteroides* ATCC 8293 | 1976.6 | 1869 | 1921 | 85.68 |
| *Listeria innocua* Clip11262 | 3093.1 | 3061 | 3145 | 89.97 |
| *Listeria monocytogenes* 4b F2365 | 2905.3 | 2821 | 2906 | 89.43 |
| *Listeria monocytogenes* EGD-e | 2944.5 | 2855 | 2940 | 90.22 |
| *Listeria monocytogenes* str. 1/2a F6854 | 2950.3 | 2967 | 3052 | 89.53 |
| *Listeria monocytogenes* str. 4b H7858 | 2972.3 | 3111 | 3191 | 89.65 |
| *Lyngbya aestuarii* | 7037.5 | 2505 | 6142 | 87.36 |
| *Magnetococcus* sp. MC-1 | 4628.7 | 3718 | 3770 | 86.10 |
| *Magnetospirillum magneticum* AMB-1 | 4967.1 | 4559 | 4763 | 88.36 |
| *Magnetospirillum magnetotacticum* MS-1 | 4503.3 | 4306 | 4357 | 89.72 |
| *Mannheimia succiniciproducens* MBEL55E | 2314.1 | 2384 | 2463 | 90.81 |
| *Marinobacter aquaeolei* VT8 | 4648.0 | 4158 | 4210 | 89.70 |
| *Mesoplasma florum* L1 | 793.2 | 683 | 716 | 94.19 |
| *Mesorhizobium loti* MAFF303099 | 7596.3 | 7281 | 7341 | 85.91 |
| *Mesorhizobium* sp. BNC1 | 4978.0 | 4480 | 4537 | 87.93 |
| *Methylobacillus flagellatus* KT | 2851.3 | 2505 | 2553 | 87.36 |
| *Methylobacillus flagellatus* KT | 2971.5 | 2759 | 2811 | 90.73 |
| *Methylococcus capsulatus* Bath | 3304.6 | 2959 | 3011 | 89.60 |
| *Moorella thermoacetica* ATCC 39073 | 2628.8 | 2523 | 2577 | 87.86 |
| *Mycobacterium avium* paratuberculosis k10 | 4829.8 | 4350 | 4399 | 91.51 |
| *Mycobacterium bovis* AF2122/97 | 4345.5 | 3953 | 4002 | 91.05 |
| *Mycobacterium leprae* TN | 3268.2 | 2720 | 2768 | 76.87 |
| *Mycobacterium* sp. JLS | 6048.4 | 5739 | 5845 | 92.00 |
| *Mycobacterium tuberculosis* CDC1551 | 4403.8 | 4189 | 4238 | 90.37 |
| *Mycobacterium tuberculosis* H37Rv | 4411.5 | 3999 | 4047 | 91.09 |
| *Mycoplasma gallisepticum* R | 996.4 | 726 | 765 | 88.43 |
| *Mycoplasma genitalium* G-37 | 580.1 | 480 | 519 | 91.47 |
| *Mycoplasma hyopneumoniae* 232 | 892.8 | 691 | 724 | 90.51 |
| *Mycoplasma hyopneumoniae* 7448 | 920.1 | 663 | 726 | 86.69 |
| *Mycoplasma hyopneumoniae* J | 897.4 | 665 | 728 | 88.41 |
| *Mycoplasma mobile* 163K | 777.1 | 635 | 666 | 91.64 |
| *Mycoplasma mycoides* SC PG1 | 1211.7 | 1016 | 1052 | 82.45 |
| *Mycoplasma penetrans* HF-2 | 1358.6 | 1037 | 1072 | 89.09 |
| *Mycoplasma pneumoniae* M129 | 816.4 | 688 | 728 | 88.69 |
| *Mycoplasma pulmonis* UAB CTIP | 963.9 | 782 | 814 | 90.73 |
| *Mycoplasma synoviae* 53 | 799.5 | 672 | 747 | 90.64 |
| *Neisseria gonorrhoeae* FA 1090 | 2153.9 | 2002 | 2069 | 79.15 |
| *Neisseria meningitidis* MC58 | 2272.4 | 2025 | 2097 | 78.70 |
| *Neisseria meningitidis* Z2491 | 2184.4 | 2121 | 2191 | 83.79 |
| *Neorickettsia sennetsu* Miyayama | 859.0 | 932 | 968 | 88.02 |
| *Nitrobacter hamburgensis* X14 | 5010.3 | 4669 | 4728 | 83.19 |
| *Nitrobacter hamburgensis* X14 | 5011.5 | 4673 | 4731 | 83.91 |
| *Nitrobacter winogradskyi* Nb-255 | 3402.1 | 3143 | 3195 | 84.90 |
| *Nitrosococcus oceani* ATCC 19707 | 3522.1 | 3132 | 3183 | 86.77 |
| *Nitrosomonas europaea* ATCC 19718 | 2812.1 | 2574 | 2619 | 88.49 |
| *Nitrosomonas eutropha* C71 | 2712.0 | 2544 | 2588 | 86.73 |
| *Nitrosospira multiformis* ATCC25196 | 3234.3 | 2827 | 2873 | 85.64 |
| *Nocardia farcinica* IFM 10152 | 6292.3 | 5936 | 5999 | 90.34 |
| *Nocardioides* sp. JS614 | 5394.1 | 4902 | 4954 | 89.35 |
| *Nostoc punctiforme* PCC73102 | 9059.2 | 7364 | 7465 | 80.91 |
| *Nostoc* sp. PCC 7120 | 7000.0 | 5366 | 5430 | 82.00 |
| *Novosphingobium aromaticivorans* DSM 12444 | 4226.6 | 3765 | 3823 | 90.79 |
| *Novosphingobium aromaticivorans* DSM 12444 | 3561.6 | 3338 | 3404 | 92.20 |
| *Oceanicaulis alexandrii* HTCC2633 | 3168.2 | 3029 | 3077 | 90.53 |
| *Oceanobacillus iheyensis* HTE831 | 3630.5 | 3496 | 3588 | 85.38 |
| *Oenococcus oeni* PSU-1 | 1782.8 | 1788 | 1834 | 84.14 |
| *Onion yellows* phytoplasma OY-M | 860.6 | 754 | 792 | 74.08 |
| *Parachlamydia* sp. UWE25 | 2414.5 | 2031 | 2075 | 82.75 |
| *Paracoccus denitrificans* PD1222 | 5175.7 | 5101 | 5157 | 91.75 |
| *Pasteurella multocida* subsp. multocida Pm70 | 2257.5 | 2014 | 2090 | 90.34 |
| *Pediococcus pentosaceus* ATCC25745 | 1814.6 | 1660 | 1722 | 86.08 |
| *Pelagibacter ubique* HTCC1062 | 1308.8 | 1354 | 1406 | 96.06 |
| *Pelobacter carbinolicus* DSM 2380 | 3662.3 | 3145 | 3205 | 86.72 |
| *Pelobacter propionicus* DSM 2379 | 4466.7 | 3828 | 3896 | 83.47 |
| *Pelodictyon luteolum* DSMZ 273(T) | 2364.8 | 2133 | 2187 | 90.55 |
| *Pelodictyon phaeoclathratiforme* BU-1 | 3000.2 | 2762 | 2814 | 86.28 |
| *Photobacterium profundum* SS9 | 6403.3 | 5480 | 5697 | 82.65 |
| *Photorhabdus luminescens* laumondii TTO1 | 5689.0 | 4905 | 5012 | 84.24 |
| *Polaromonas naphthalenivorans* CJ2 | 5340.2 | 4973 | 5022 | 88.90 |
| *Polaromonas* sp. JS666 | 5912.3 | 5662 | 5709 | 88.97 |
| *Polaromonas* sp. JS666 | 5898.7 | 5569 | 5616 | 88.60 |
| *Porphyromonas gingivalis* W83 | 2343.5 | 1909 | 1974 | 83.33 |
| *Prochlorococcus marinus* MED4 | 1658.0 | 1716 | 1756 | 88.48 |
| *Prochlorococcus marinus* MIT 9313 | 2410.9 | 2273 | 2324 | 82.28 |
| *Prochlorococcus marinus* str. MIT 9211 | 1839.0 | 2123 | 2168 | 89.00 |
| *Prochlorococcus marinus* str. MIT 9301 | 1641.9 | 1907 | 1963 | 90.00 |
| *Prochlorococcus marinus* str. MIT 9303 | 2682.7 | 2997 | 3136 | 84.00 |
| *Prochlorococcus marinus* str. MIT 9312 | 1709.0 | 1809 | 1855 | 89.00 |
| Prochlorococcus marinus str. NATL2A | 1843.0 | 1890 | 1938 | 85.00 |
| *Prochlorococcus marinus* subsp. marinus CCMP1375 | 1751.1 | 1882 | 1926 | 89.13 |
| *Prochlorococcus marinus* subsp. marinus str. SS120 (CCMP1375) | 1751.0 | 1882 | 1930 | 88.00 |
| *Prochlorococcus marinus* subsp. pastoris str. CCMP1986(MED4) | 1668.0 | 1712 | 1760 | 87.00 |
| *Prochlorococcus* sp. CC9902 (coastal) | 2234.8 | 2321 | 2372 | 90.31 |
| *Propionibacterium acnes* KPA171202 | 2560.3 | 2297 | 2351 | 89.87 |
| *Prosthecochloris aestuarii* SK413 | 2563.2 | 2315 | 2359 | 87.41 |
| *Pseudoalteromonas atlantica* T6c | 5095.0 | 4231 | 4297 | 86.68 |
| *Pseudoalteromonas haloplanktis* TAC 125 | 3850.3 | 3487 | 3621 | 88.39 |
| *Pseudomonas aeruginosa* PAO1 | 6264.4 | 5566 | 5642 | 89.59 |
| *Pseudomonas aeruginosa* UCBPP-PA14 | 6524.1 | 5688 | 5754 | 85.93 |
| *Pseudomonas fluorescens* Pf-5 | 7074.9 | 6137 | 6295 | 89.06 |
| *Pseudomonas fluorescens* PfO-1 | 6438.4 | 5745 | 5837 | 90.42 |
| *Pseudomonas putida* F1 | 5925.1 | 5251 | 5341 | 89.13 |
| *Pseudomonas putida* KT2440 | 6181.9 | 5350 | 5445 | 87.19 |
| *Pseudomonas syringae* B728a | 6093.7 | 5137 | 5217 | 88.50 |
| *Pseudomonas syringae* phaseolicola 1448A | 6112.4 | 5170 | 5312 | 84.15 |
| *Pseudomonas syringae* pv. tomato DC3000 | 6538.3 | 5608 | 5688 | 85.38 |
| *Psychrobacter arcticum* 273-4 | 2650.7 | 2147 | 2208 | 82.06 |
| *Psychrobacter cryohalolentis* K5 | 3081.1 | 2485 | 2538 | 83.08 |
| *Psychrobacter cryohalolentis* K5 | 3101.1 | 2515 | 2575 | 83.80 |
| *Psychrobacter* sp. PRwf-1 | 2953.1 | 2350 | 2394 | 83.77 |
| *Ralstonia solanacearum* GMI1000 | 5810.9 | 5120 | 5189 | 87.78 |
| *Rhizobium etli* CFN 42 | 4382.0 | 4043 | 4126 | 91.89 |
| *Rhizobium etli* CFN 42 | 6159.0 | 5627 | 5686 | 86.42 |
| *Rhodobacter sphaeroides* 2.4.1 | 4603.1 | 4304 | 4367 | 88.28 |
| *Rhodobacter sphaeroides* ATCC 17025 | 4479.4 | 4248 | 4296 | 88.68 |
| *Rhodobacter sphaeroides* ATCC 17029 | 4462.0 | 3971 | 4019 | 88.28 |
| *Rhodobacter sphaeroides* ATCC 17029 (chr1+2) | 4366.8 | 4025 | 4135 | 88.16 |
| *Rhodoferax ferrireducens* DSM 15236 | 4926.7 | 4378 | 4427 | 88.56 |
| *Rhodoferax ferrireducens* DSM 15236 | 4969.8 | 4495 | 4546 | 90.32 |
| *Rhodopirellula baltica* SH 1 | 7145.6 | 7321 | 7403 | 94.95 |
| *Rhodopirellula baltica* SH1 | 7146.0 | 7325 | 7404 | 94.00 |
| *Rhodopseudomonas palustris* BisA53 | 5502.4 | 4913 | 4969 | 86.33 |
| *Rhodopseudomonas palustris* BisB18 | 5505.0 | 4949 | 5007 | 86.01 |
| *Rhodopseudomonas palustris* BisB18 | 5513.8 | 4943 | 4999 | 86.23 |
| *Rhodopseudomonas palustris* BisB5 | 4890.4 | 4385 | 4443 | 87.09 |
| *Rhodopseudomonas palustris* BisB5 | 4892.7 | 4418 | 4475 | 87.24 |
| *Rhodopseudomonas palustris* CGA009 | 5467.6 | 4840 | 4897 | 87.53 |
| *Rhodopseudomonas palustris* HaA2 | 5331.5 | 4683 | 4736 | 87.62 |
| *Rhodopseudomonas palustris* HaA2 | 5331.7 | 4712 | 4765 | 87.58 |
| *Rhodoseudomonas palustris* CGA009 | 5459.0 | 4813 | 4891 | 87.00 |
| *Rhodospirillum rubrum* ATCC 11170 | 4406.6 | 3850 | 3917 | 88.82 |
| *Rickettsia akari* Hartford | 1231.1 | 1217 | 1253 | 77.43 |
| *Rickettsia bellii* RML369-C | 1522.1 | 1429 | 1466 | 85.39 |
| *Rickettsia conorii* Malish 7 | 1268.8 | 1374 | 1410 | 80.84 |
| *Rickettsia felis* URRWXCal2 | 1587.2 | 1512 | 1548 | 83.82 |
| *Rickettsia prowazekii* Madrid E | 1111.5 | 834 | 870 | 76.03 |
| *Rickettsia rickettsii* | 1257.7 | 1311 | 1346 | 78.50 |
| *Rickettsia sibirica* 246 | 1250.0 | 1234 | 1270 | 78.13 |
| *Rickettsia typhi* Wilmington | 1111.5 | 838 | 874 | 75.92 |
| *Roseovarius nubinhibens* ISM | 3668.7 | 3547 | 3597 | 89.58 |
| *Rubrivivax gelatinosus* PM1 | 4643.7 | 4475 | 4548 | 91.89 |
| *Rubrobacter xylanophilus* DSM 9941 | 3299.4 | 2990 | 3039 | 86.62 |
| *Saccharophagus degradans* 2-40 | 5057.5 | 4017 | 4064 | 87.03 |
| *Saccharophagus degradans* 2-40 | 5057.5 | 4017 | 4064 | 86.99 |
| *Saccharopolyspora erythraea* NRRL 2338 | 8212.8 | 7191 | 7264 | 84.00 |
| *Salinibacter ruber* DSM 13855 | 3587.3 | 2833 | 2880 | 84.88 |
| *Salmonella enterica* choleraesuis SC-B67 | 4944.0 | 4666 | 4773 | 84.26 |
| *Salmonella enterica* CT18 | 4809.0 | 4395 | 4711 | 83.00 |
| *Salmonella enterica* Paratyphi-A SARB42 | 4585.2 | 4093 | 4197 | 83.22 |
| *Salmonella enterica* Typhi CT18 | 5133.7 | 4980 | 5082 | 88.07 |
| *Salmonella enterica* Typhi Ty2 | 4792.0 | 4323 | 4423 | 83.08 |
| *Salmonella typhimurium* LT2 | 4951.4 | 4554 | 4692 | 87.28 |
| *Shewanella amazonensis* SB2B | 4264.5 | 3577 | 3702 | 87.83 |
| *Shewanella baltica* OS155 | 5084.3 | 4334 | 4458 | 82.56 |
| *Shewanella baltica* OS155 (chr) | 5127.4 | 4307 | 4521 | 83.00 |
| *Shewanella denitrificans* OS217 | 4491.5 | 3722 | 3840 | 84.27 |
| *Shewanella denitrificans* OS217 | 4545.9 | 3778 | 3896 | 85.68 |
| *Shewanella frigidimarina* NCMB400 | 4782.4 | 4002 | 4124 | 84.41 |
| *Shewanella loihica* PV-4 | 4602.6 | 3859 | 3993 | 85.00 |
| *Shewanella oneidensis* MR-1 | 5131.4 | 4778 | 4908 | 83.89 |
| *Shewanella putrefaciens* CN-32 | 4577.7 | 3981 | 4095 | 85.15 |
| *Shewanella* sp. ANA-3 | 5100.7 | 4241 | 4353 | 85.99 |
| *Shewanella* sp. MR-4 | 4579.2 | 3852 | 3928 | 85.44 |
| *Shewanella* sp. MR-7 | 4546.4 | 3824 | 3920 | 85.56 |
| *Shewanella* sp. PV-4 | 4474.5 | 3704 | 3811 | 85.72 |
| *Shewanella* sp. W3-18-1 | 4754.0 | 4117 | 4241 | 84.35 |
| *Shigella boydii* sv4 Sb227 | 4646.5 | 4290 | 4403 | 81.10 |
| *Shigella dysenteriae* sv1 Sd197 | 4552.0 | 4500 | 4607 | 77.88 |
| *Shigella flexneri* 2a 2457T | 4599.4 | 4073 | 4195 | 78.05 |
| *Shigella flexneri* 2a 301 | 4828.8 | 4703 | 4822 | 86.98 |
| *Shigella sonnei* Ss046 | 5039.7 | 4462 | 4581 | 81.02 |
| *Silicibacter pomeroyi* DSS-3 | 4601.1 | 4252 | 4314 | 89.92 |
| *Silicibacter* sp. TM1040 | 4198.3 | 3860 | 3932 | 88.15 |
| *Sinorhizobium meliloti* 1021 | 6691.7 | 6205 | 6270 | 86.25 |
| *Sinorhizobium meliloti* 1021 | 6691.7 | 6205 | 6269 | 86.25 |
| *Solibacter usitatus* Ellin6076 | 9949.9 | 8098 | 8157 | 90.92 |
| *Sphingopyxis alaskensis* RB2256 | 3343.4 | 3199 | 3247 | 91.37 |
| Staphylococcus aureus bovine RF122 | 2742.5 | 2589 | 2665 | 84.81 |
| *Staphylococcus* *aureus* COL | 2813.9 | 2618 | 2690 | 83.69 |
| *Staphylococcus aureus* JH1 | 2879.6 | 2681 | 2737 | 83.69 |
| *Staphylococcus aureus* JH9 | 2862.9 | 2673 | 2674 | 83.53 |
| *Staphylococcus aureus* MRSA252 | 2902.6 | 2744 | 2820 | 84.79 |
| *Staphylococcus aureus* MSSA476 | 2820.5 | 2643 | 2722 | 84.81 |
| *Staphylococcus aureus* Mu50 | 2903.6 | 2733 | 2809 | 84.59 |
| *Staphylococcus aureus* MW2 | 2841.1 | 2659 | 2738 | 84.60 |
| *Staphylococcus aureus* N315 | 2839.5 | 2624 | 2702 | 84.36 |
| *Staphylococcus aureus* NCTC 832 | 2821.4 | 2892 | 2969 | 85.71 |
| *Staphylococcus* *aureus* USA300 | 2917.5 | 2604 | 2673 | 82.94 |
| *Staphylococcus epidermidis* ATCC 12228 | 2564.6 | 2485 | 2561 | 84.13 |
| *Staphylococcus epidermidis* RP62A | 2643.8 | 2526 | 2606 | 83.70 |
| *Staphylococcus haemolyticus* JCSC1435 | 2685.0 | 2678 | 2813 | 86.93 |
| *Staphylococcus saprophyticus* saprophyticus ATCC 15305 | 2577.9 | 2514 | 2595 | 84.70 |
| *Streptococcus agalactiae* 2603V/R | 2160.3 | 2124 | 2225 | 88.14 |
| *Streptococcus agalactiae* Ia A909 | 2127.8 | 1996 | 2097 | 87.85 |
| *Streptococcus agalactiae* NEM316 | 2211.5 | 2134 | 2235 | 90.17 |
| *Streptococcus mutans* UA159 | 2030.9 | 1960 | 2040 | 87.06 |
| *Streptococcus pneumoniae* R6 | 2038.6 | 2043 | 2113 | 87.57 |
| *Streptococcus pneumoniae* TIGR4 | 2160.8 | 2094 | 2164 | 83.54 |
| *Streptococcus pyogenes* M1 GAS | 1852.4 | 1696 | 1774 | 85.34 |
| *Streptococcus pyogenes* M1 MGAS5005 | 1838.6 | 1865 | 2017 | 88.22 |
| *Streptococcus pyogenes* M28 MGAS6180 | 1897.6 | 1894 | 2044 | 88.48 |
| *Streptococcus pyogenes* MGAS10394 | 1899.9 | 1886 | 1973 | 88.65 |
| *Streptococcus pyogenes* MGAS315 | 1900.5 | 1865 | 1946 | 87.23 |
| *Streptococcus pyogenes* MGAS8232 | 1895.0 | 1845 | 1930 | 86.70 |
| *Streptococcus pyogenes* SSI-1 | 1894.3 | 1861 | 1930 | 86.13 |
| *Streptococcus suis* 89/1591 | 1978.2 | 1896 | 1938 | 86.10 |
| *Streptococcus thermophilus* CNRZ 1066 | 1796.2 | 1915 | 2000 | 85.47 |
| *Streptococcus thermophilus* LMD-9 | 1842.1 | 1850 | 1919 | 76.15 |
| *Streptococcus thermophilus* LMG 18311 | 1796.8 | 1889 | 1974 | 85.52 |
| *Streptomyces avermitilis* MA-4680 | 9119.9 | 7673 | 7761 | 86.39 |
| *Streptomyces coelicolor* A3(2) | 9054.8 | 8215 | 8299 | 89.13 |
| *Sulfitobacter* sp. EE-36 | 3547.2 | 3474 | 3529 | 90.89 |
| *Sulfitobacter* sp. NAS-14.1 | 4002.1 | 3962 | 4014 | 89.92 |
| *Symbiobacterium thermophilum* IAM 14863 | 3566.1 | 3337 | 3454 | 88.01 |
| *Synechococcus elongatus* BP-1 | 2593.9 | 2475 | 2520 | 89.80 |
| *Synechococcus elongatus* PCC7942 | 2695.0 | 2611 | 2664 | 88.00 |
| *Synechococcus* sp. CC9605 | 2511.0 | 2638 | 2749 | 86.00 |
| *Synechococcus* sp. CC9902 | 2235.0 | 2304 | 2355 | 89.00 |
| *Synechococcus* sp. JA-2-3B'a(2-13) | 3047.0 | 2862 | 2942 | 85.00 |
| *Synechococcus* sp. JA-3-3Ab | 2933.0 | 2760 | 2897 | 84.00 |
| *Synechococcus* sp. PCC 6301 | 2696.3 | 2525 | 2576 | 87.98 |
| *Synechococcus* sp. RS9917 | 2580.0 | 2770 | 2820 | 92.00 |
| *Synechococcus* sp. WH 5701 | 3043.0 | 3346 | 3401 | 87.00 |
| *Synechococcus* sp. WH 7805 | 2620.0 | 2883 | 2394 | 90.00 |
| *Synechococcus* sp. WH8102 | 2434.4 | 2526 | 2576 | 90.45 |
| *Synechocystis* sp. PCC 6803 | 3947.0 | 3564 | 3614 | 86.69 |
| *Syntrophobacter fumaroxidans* MPOB | 4848.8 | 4031 | 4084 | 82.96 |
| *Syntrophomonas wolfei* Goettingen | 2845.8 | 2466 | 2524 | 83.33 |
| *Syntrophus aciditrophicus* | 3179.3 | 3168 | 3219 | 88.68 |
| *Thermoanaerobacter ethanolicus* 39E | 2282.7 | 2254 | 2311 | 87.84 |
| *Thermoanaerobacter tengcongensis* MB4 | 2689.4 | 2588 | 2652 | 87.42 |
| *Thermobifida fusca* YX | 3642.2 | 3117 | 3182 | 85.74 |
| *Thermotoga maritima* MSB8 | 1860.7 | 1846 | 1895 | 93.88 |
| *Thermus thermophilus* HB27 | 2127.5 | 2210 | 2263 | 94.77 |
| *Thermus thermophilus* HB8 | 1859.1 | 1988 | 2042 | 95.70 |
| *Thiobacillus denitrificans* sp. ATCC 25259 | 2909.8 | 2827 | 2876 | 92.78 |
| *Thiomicrospira crunogena* XCL-2 | 2427.7 | 2201 | 2254 | 90.63 |
| *Thiomicrospira denitrificans* ATCC 33889 | 2201.6 | 2104 | 2160 | 93.80 |
| *Treponema denticola* ATCC 35405 | 2843.2 | 2767 | 2817 | 91.93 |
| *Treponema pallidum* Nichols | 1138.0 | 1031 | 1082 | 93.15 |
| *Trichodesmium erythraeum* IMS101 | 8191.4 | 4759 | 4801 | 58.12 |
| *Tropheryma whipplei* TW08/27 | 925.9 | 784 | 838 | 85.61 |
| *Tropheryma whipplei* Twist | 927.3 | 808 | 861 | 86.34 |
| *Ureaplasma parvum* serovar 3 ATCC 700970 | 751.7 | 611 | 647 | 92.43 |
| *Vibrio cholerae* O1 biovar eltor N16961 | 4033.5 | 3828 | 3951 | 87.42 |
| *Vibrio fischeri* ES114 | 4238.2 | 3747 | 3903 | 87.95 |
| *Vibrio parahaemolyticus* RIMD 2210633 | 5165.8 | 4832 | 4992 | 87.86 |
| *Vibrio* *vulnificus* CMCP6 | 5126.8 | 4537 | 4676 | 85.73 |
| *Vibrio vulnificus* YJ016 | 5260.1 | 5028 | 5168 | 89.25 |
| *Wigglesworthia glossinidia* endosymbiont of Glossina | 697.7 | 611 | 651 | 88.47 |
| *Wolbachia endosymbiont* of Drosophila ananassae | 1440.8 | 1802 | 1833 | 75.78 |
| *Wolbachia endosymbiont* of Drosophila simulans | 1063.1 | 760 | 782 | 65.02 |
| *Wolbachia endosymbiont* strain TRS of Brugia malayi | 1080.1 | 805 | 842 | 67.60 |
| *Wolbachia pipientis* wMel | 1267.8 | 1195 | 1232 | 80.59 |
| *Wolinella succinogenes* DSM 1740 | 2110.4 | 2044 | 2090 | 94.46 |
| *Xanthobacter autotrophicus* Py2 | 5580.9 | 5041 | 5042 | 88.19 |
| *Xanthomonas axonopodis* citri 306 | 5274.2 | 4427 | 4487 | 86.01 |
| *Xanthomonas campestris* campestris 8004 | 5148.7 | 4273 | 4389 | 84.89 |
| *Xanthomonas campestris* campestris ATCC 33913 | 5076.2 | 4181 | 4241 | 84.80 |
| *Xanthomonas campestris* vesicatoria 85-10 | 5420.2 | 4726 | 4786 | 87.35 |
| *Xanthomonas oryzae* KACC10331 | 4941.4 | 4637 | 4697 | 85.98 |
| *Xanthomonas oryzae* MAFF 311018 | 4940.2 | 4372 | 4431 | 84.15 |
| *Xylella fastidiosa* 9a5c | 2731.8 | 2832 | 2888 | 83.64 |
| *Xylella fastidiosa* Ann-1 | 2622.3 | 2359 | 2411 | 80.73 |
| *Xylella fastidiosa* Dixon | 5118.9 | 4662 | 4757 | 79.14 |
| *Xylella fastidiosa* Temecula1 | 2521.1 | 2036 | 2091 | 78.32 |
| *Yersinia pestis* biovar Medievalis 91001 | 4803.2 | 4142 | 4237 | 82.08 |
| *Yersinia pestis* CO92 | 4829.9 | 4217 | 4307 | 84.23 |
| *Yersinia pestis* KIM | 4600.8 | 4090 | 4187 | 83.19 |
| *Yersinia pseudotuberculosis* IP 32953 | 4840.9 | 4116 | 4226 | 84.27 |
| *Zymomonas mobilis* ZM4 | 2056.4 | 1998 | 2086 | 86.72 |
|  |  |  |  |  |
| **Archaea** |  |  |  |  |
| *Aeropyrum pernix* K1 | 1669.7 | 2694 | 2751 | 89.17 |
| *Aeropyrum pernix* K1 | 1669.7 | 2763 | 2815 | 90.44 |
| *Archaeoglobus fulgidus* DSM 4304 | 2178.4 | 2407 | 2456 | 91.20 |
| *Archaeoglobus fulgidus* VC-16 | 2178.4 | 2466 | 2515 | 93.62 |
| *Cenarchaeum symbiosum* | 2023.7 | 2011 | 2059 | 91.52 |
| *Ferroplasma acidarmanus* fer1 | 1971.4 | 1695 | 1742 | 80.26 |
| *Haloarcula marismortui* ATCC 43049 | 4274.6 | 4240 | 4300 | 84.96 |
| *Haloarcula marismortui* ATCC 43049 | 4274.6 | 4322 | 4382 | 86.39 |
| *Halobacterium salinarium* NRC-1 | 2571.0 | 2675 | 2726 | 87.41 |
| *Halobacterium salinarum* NRC-1 | 2571.0 | 2605 | 2657 | 85.20 |
| *Methanobacterium thermoautotrophicum* Delta H | 1751.4 | 1846 | 1891 | 91.12 |
| *Methanocaldococcus jannaschii* DSM 2661 | 1739.9 | 1770 | 1813 | 87.23 |
| *Methanocaldococcus jannaschii* DSM 2661 | 1739.9 | 1789 | 1832 | 88.75 |
| *Methanococcoides burtonii* DSM 6242 | 2575.0 | 2434 | 2495 | 85.69 |
| *Methanococcoides burtonii* DSM6242 | 2597.4 | 2490 | 2551 | 84.21 |
| *Methanococcus maripaludis* C5 | 1780.8 | 1813 | 1880 | 86.00 |
| *Methanococcus maripaludis* S2 | 1661.1 | 1722 | 1770 | 89.87 |
| *Methanococcus maripaludis* S2 | 1661.1 | 1732 | 1780 | 90.03 |
| *Methanoculleus marisnigri* | 2460.2 | 2418 | 2466 | 87.51 |
| *Methanoculleus marisnigri* JR1 | 2478.1 | 2489 | 2555 | 87.00 |
| *Methanopyrus kandleri* AV19 | 1695.0 | 1691 | 1734 | 89.09 |
| *Methanopyrus kandleri* AV19 | 1695.0 | 1727 | 1765 | 90.24 |
| *Methanosarcina acetivorans* C2A | 5751.5 | 4540 | 4609 | 74.28 |
| *Methanosarcina acetivorans* C2A | 5751.5 | 4721 | 4731 | 76.77 |
| *Methanosarcina barkeri* Fusaro | 4873.8 | 3759 | 3830 | 71.46 |
| *Methanosarcina mazei* Go1 | 4096.3 | 3371 | 3438 | 75.83 |
| *Methanosarcina mazei* Go1 | 4096.3 | 3398 | 3465 | 76.51 |
| *Methanosphaera stadtmanae* DSM 3091 | 1767.4 | 1534 | 1586 | 85.20 |
| *Methanospirillum hungatei* JF-1 | 3535.0 | 3307 | 3369 | 87.10 |
| *Methanospiriilum hungatei* JF-1 | 3544.7 | 3238 | 3303 | 88.62 |
| *Methanothermobacter thermautotrophicus* Delta H | 1751.4 | 1869 | 1915 | 90.91 |
| *Methaosaeta thermophila* PT | 1861.6 | 1718 | 1761 | 84.05 |
| *Nanoarchaeum equitans* Kin4-M | 490.9 | 536 | 579 | 93.39 |
| *Nanoarchaeum equitans* Kin4-M | 490.9 | 556 | 573 | 93.07 |
| *Natronomonas pharaonis* DSM 2160 | 2749.7 | 2843 | 2892 | 90.61 |
| *Picrophilus torridus* DSM 9790 | 1545.9 | 1535 | 1628 | 91.92 |
| *Picrophilus torridus* DSM 9790 | 1545.9 | 1551 | 1598 | 92.41 |
| *Pyrobaculum aerophilum* IM2 | 2222.4 | 2605 | 2655 | 88.78 |
| *Pyrobaculum aerophilum* IM2 | 2222.4 | 2575 | 2614 | 89.24 |
| *Pyrobaculum calidifontis* JCM 11548 | 2009.3 | 2149 | 2200 | 90.00 |
| *Pyrococcus abyssi* GE5 | 1765.1 | 1784 | 1881 | 92.04 |
| *Pyrococcus abyssi* GE5 | 1768.6 | 1904 | 1955 | 93.78 |
| *Pyrococcus furiosus* DSM 3638 | 1908.3 | 2065 | 2115 | 91.38 |
| *Pyrococcus furiosus* DSM 3638 | 1908.3 | 2069 | 2171 | 92.66 |
| *Pyrococcus horikoshii* OT3 | 1738.5 | 2061 | 2112 | 91.07 |
| *Pyrococcus horikoshii* OT3 | 1738.5 | 2196 | 2297 | 93.31 |
| *Staphylothermus marinus* F1 | 1570.5 | 1570 | 1646 | 86.00 |
| *Sulfolobus acidocaldarius* DSM 639 | 2226.0 | 2223 | 2322 | 85.31 |
| *Sulfolobus acidocaldarius* DSM 639 | 2226.0 | 2285 | 2336 | 87.67 |
| *Sulfolobus solfataricus* P2 | 2992.2 | 2994 | 3050 | 84.32 |
| *Sulfolobus solfataricus* P2 | 2992.2 | 3048 | 3104 | 86.43 |
| *Sulfolobus tokodaii* 7 | 2694.8 | 2826 | 2878 | 83.36 |
| *Sulfolobus tokodaii* str. 7 | 2694.8 | 2915 | 2964 | 85.52 |
| *Thermococcus kodakaraensis* KOD1 | 2088.7 | 2306 | 2358 | 92.05 |
| *Thermococcus kodakaraensis* KOD1 | 2088.7 | 2297 | 2349 | 92.09 |
| *Thermofilum pendens* | 1811.4 | 1892 | 1932 | 90.99 |
| *Thermoplasma acidophilum* DSM 1728 | 1564.9 | 1478 | 1549 | 87.02 |
| *Thermoplasma acidophilum* DSM 1728 | 1564.9 | 1527 | 1575 | 88.51 |
| *Thermoplasma volcanium* GSS1 | 1584.8 | 1526 | 1575 | 86.19 |
| *Thermoplasma volcanium* GSS1 | 1584.8 | 1561 | 1610 | 88.26 |
|  |  |  |  |  |
| **Virus** |  |  |  |  |
| Acholeplasma phage L2 | 12.0 | 14 | 14 | 81.31 |
| Acholeplasma phage MV-L1 | 4.5 | 4 | 4 | 46.60 |
| Actinoplanes phage phiAsp2 | 58.6 | 76 | 76 | 92.23 |
| Aeromonas phage 31 | 173.0 | 247 | 263 | 92.84 |
| Bacillus clarkii bacteriophage BCJA1c | 41.1 | 58 | 58 | 92.66 |
| Bacillus phage GA-1 | 21.1 | 35 | 35 | 92.46 |
| Bacillus phage phi29 | 19.4 | 27 | 27 | 93.88 |
| Bacillus thuringiensis bacteriophage Bam35c | 14.9 | 32 | 32 | 93.49 |
| Bacillus thuringiensis phage GIL16c | 14.8 | 31 | 31 | 94.09 |
| Bacteriophage 11b | 36.1 | 65 | 65 | 90.15 |
| Bacteriophage 187 | 39.6 | 77 | 77 | 93.55 |
| Bacteriophage 2638A | 41.3 | 57 | 57 | 92.68 |
| Bacteriophage 29 | 42.8 | 75 | 75 | 92.15 |
| Bacteriophage 37 | 43.7 | 77 | 77 | 95.58 |
| Bacteriophage 3A | 43.1 | 67 | 67 | 93.95 |
| Bacteriophage 42e | 45.9 | 79 | 79 | 93.38 |
| Bacteriophage 44RR2.8t | 173.6 | 252 | 269 | 93.28 |
| Bacteriophage 47 | 44.8 | 72 | 72 | 93.99 |
| Bacteriophage 52A | 41.7 | 65 | 65 | 91.64 |
| Bacteriophage 53 | 43.9 | 79 | 79 | 93.42 |
| Bacteriophage 55 | 41.9 | 77 | 77 | 93.37 |
| Bacteriophage 66 | 18.2 | 27 | 27 | 91.85 |
| Bacteriophage 69 | 42.7 | 76 | 76 | 93.02 |
| Bacteriophage 71 | 43.1 | 72 | 72 | 94.55 |
| Bacteriophage 77 | 41.7 | 69 | 69 | 92.12 |
| Bacteriophage 85 | 44.3 | 78 | 78 | 91.80 |
| Bacteriophage 88 | 43.2 | 72 | 72 | 94.35 |
| Bacteriophage 92 | 42.4 | 74 | 74 | 95.17 |
| Bacteriophage 933W | 61.7 | 80 | 83 | 88.07 |
| Bacteriophage 96 | 43.6 | 79 | 79 | 91.98 |
| Bacteriophagie A118 | 40.8 | 72 | 72 | 94.19 |
| Bacteriophage Aaphi23 | 43.0 | 66 | 66 | 92.42 |
| Bacteriophage Aeh1 | 233.2 | 352 | 377 | 94.04 |
| Bacteriophage AP205 | 4.3 | 4 | 4 | 90.96 |
| Bacteriophage B103 | 18.6 | 17 | 17 | 84.76 |
| Bacteriophage B3 | 38.4 | 59 | 59 | 97.13 |
| Bacteriophage bIL170 | 31.8 | 64 | 65 | 91.44 |
| Bacteriophage bIL285 | 35.5 | 62 | 62 | 93.03 |
| Bacteriophage bIL286 | 41.8 | 61 | 61 | 93.09 |
| Bacteriophage bIL309 | 36.9 | 56 | 56 | 89.47 |
| Bacteriophage bIL310 | 15.0 | 29 | 29 | 77.29 |
| Bacteriophage bIL311 | 14.5 | 22 | 23 | 61.98 |
| Bacteriophage bIL312 | 15.2 | 27 | 28 | 75.55 |
| Bacteriophage D3112 | 37.6 | 55 | 55 | 93.84 |
| Bacteriophage EJ-1 | 42.9 | 73 | 73 | 92.25 |
| Bacteriophage EW | 45.3 | 77 | 77 | 92.04 |
| Bacteriophage Felix 01 | 86.2 | 247 | 269 | 92.91 |
| Bacteriophage G1 | 138.7 | 214 | 218 | 88.69 |
| Bacteriophage HK620 | 38.3 | 58 | 58 | 88.62 |
| Bacteriophage IN93 | 19.6 | 35 | 35 | 83.53 |
| Bacteriophage K139 | 33.1 | 44 | 44 | 92.10 |
| Bacteriophage KS7 | 40.8 | 56 | 56 | 74.94 |
| Bacteriophage KVP40 | 244.8 | 381 | 410 | 91.06 |
| Bacteriophage L-413C | 30.7 | 40 | 40 | 93.17 |
| Bacteriophage L5 | 2.4 | 8 | 8 | 78.97 |
| Bacteriophage lambda | 48.5 | 71 | 71 | 87.83 |
| Bacteriophage Mx8 | 49.5 | 86 | 86 | 93.40 |
| Bacteriophage P27 | 42.6 | 58 | 60 | 89.84 |
| Bacteriophage P4 | 11.6 | 14 | 14 | 84.25 |
| Bacteriophage phBC6A51 | 61.4 | 75 | 75 | 82.74 |
| Bacteriophage phBC6A52 | 38.5 | 49 | 49 | 80.07 |
| Bacteriophage phi AT3 | 39.2 | 55 | 55 | 89.65 |
| Bacteriophage phi CTX | 35.6 | 47 | 47 | 91.27 |
| Bacteriophage phi ETA | 43.1 | 66 | 66 | 92.79 |
| Bacteriophage phi JL001 | 63.6 | 90 | 90 | 95.32 |
| Bacteriophage phi LC3 | 32.2 | 51 | 51 | 92.46 |
| Bacteriophage phi1026b | 54.9 | 83 | 83 | 94.00 |
| Bacteriophage phi-105 | 39.3 | 51 | 51 | 89.23 |
| Bacteriophage phi-12 | 13.2 | 15 | 15 | 85.87 |
| Bacteriophage phi3626 | 33.5 | 50 | 50 | 92.54 |
| Bacteriophage phi-8 | 15.0 | 16 | 16 | 89.33 |
| Bacteriophage phi-BT1 | 41.8 | 55 | 58 | 88.36 |
| Bacteriophage phi-C31 | 41.5 | 53 | 55 | 90.01 |
| Bacteriophage phiE125 | 53.4 | 71 | 71 | 89.70 |
| Bacteriophage phig1e | 42.3 | 50 | 52 | 76.25 |
| Bacteriophage phiKMV | 42.5 | 48 | 48 | 90.81 |
| Bacteriophage phiKO2 | 51.6 | 64 | 64 | 92.50 |
| Bacteriophage phiMFV1 | 15.1 | 17 | 17 | 91.61 |
| Bacteriophage phiYeO3-12 | 39.6 | 59 | 59 | 91.36 |
| Bacteriophage PSP3 | 30.6 | 42 | 42 | 91.69 |
| Bacteriophage PT1028 | 15.6 | 22 | 22 | 77.42 |
| Bacteriophage PY54 | 46.3 | 67 | 67 | 89.84 |
| Bacteriophage r1t | 33.3 | 50 | 50 | 91.26 |
| Bacteriophage RM 378 | 129.9 | 146 | 147 | 93.44 |
| Bacteriophage ROSA | 43.2 | 74 | 74 | 92.86 |
| Bacteriophage sk1 | 28.5 | 56 | 57 | 89.53 |
| Bacteriophage SPBc2 | 134.4 | 185 | 185 | 86.22 |
| Bacteriophage S-PM2 | 196.3 | 239 | 264 | 94.01 |
| Bacteriophage SPP1 | 44.0 | 106 | 106 | 92.53 |
| Bacteriophage Tuc2009 | 38.3 | 56 | 56 | 92.12 |
| Bacteriophage Vf12 | 8.0 | 7 | 7 | 55.02 |
| Bacteriophage Vf33 | 8.0 | 7 | 7 | 55.02 |
| Bacteriophage VfO3K6 | 8.8 | 10 | 10 | 79.52 |
| Bacteriophage VfO4K68 | 6.9 | 8 | 8 | 77.62 |
| Bacteriophage VSKK | 6.8 | 6 | 6 | 49.49 |
| Bacteriophage VT2-Sa | 60.9 | 83 | 86 | 88.79 |
| Bacteriophage VWB | 49.2 | 61 | 61 | 82.15 |
| Bacteriophage WPhi | 32.7 | 44 | 44 | 92.64 |
| Bacteriophage X2 | 43.4 | 77 | 77 | 92.85 |
| Bordetella phage BIP-1 | 42.6 | 48 | 48 | 95.65 |
| Bordetella phage BMP-1 | 42.7 | 47 | 47 | 94.84 |
| Bordetella phage BPP-1 | 42.5 | 49 | 49 | 96.37 |
| Burkholderia cenocepacia phage Bcep1 | 48.2 | 71 | 71 | 91.74 |
| Burkholderia cenocepacia phage BcepB1A | 47.4 | 58 | 58 | 89.26 |
| Burkholderia cenocepacia phage BcepMu | 36.7 | 53 | 53 | 94.23 |
| Burkholderia cepacia complex phage BcepC6B | 42.4 | 46 | 46 | 92.67 |
| Burkholderia cepacia phage Bcep22 | 63.9 | 78 | 79 | 91.44 |
| Burkholderia cepacia phage Bcep43 | 48.0 | 65 | 66 | 92.81 |
| Burkholderia cepacia phage Bcep781 | 48.2 | 66 | 67 | 92.83 |
| Burkholderia cepacia phage BcepNazgul | 58.1 | 75 | 75 | 90.26 |
| Chlamydia phage 2 | 4.6 | 8 | 8 | 90.29 |
| Chlamydia phage 3 | 4.6 | 8 | 8 | 89.09 |
| Chlamydia pneumoniae phage CPAR39 | 4.5 | 7 | 7 | 87.51 |
| Chlamydia psittaci bacteriophage chp1 | 4.9 | 12 | 12 | 68.59 |
| Coliphage alpha3 | 6.1 | 10 | 10 | 83.77 |
| Coliphage ID11 | 5.6 | 11 | 11 | 94.94 |
| Coliphage phiK | 6.1 | 10 | 10 | 83.84 |
| Coliphage phiX174 | 5.4 | 11 | 11 | 95.08 |
| Cyanophage P60 | 47.9 | 80 | 80 | 81.71 |
| Cyanophage P-SSM2 | 252.4 | 329 | 330 | 94.73 |
| Cyanophage P-SSM4 | 178.2 | 198 | 198 | 92.61 |
| Cyanophage P-SSP7 | 45.0 | 53 | 53 | 88.76 |
| Ehux virus | 407.0 | 472 | 477 | 81.71 |
| Enterobacteria phage 186 | 30.6 | 46 | 47 | 93.29 |
| Enterobacteria phage epsilon15 | 39.7 | 49 | 49 | 92.39 |
| Enterobacteria phage FI | 4.3 | 4 | 4 | 95.42 |
| Enterobacteria phage fr | 3.6 | 4 | 4 | 90.80 |
| Enterobacteria phage G4 | 5.6 | 11 | 11 | 94.94 |
| Enterobacteria phage GA | 3.5 | 3 | 3 | 91.32 |
| Enterobacteria phage HK022 | 40.8 | 57 | 57 | 84.17 |
| Enterobacteria phage HK97 | 39.7 | 61 | 61 | 88.50 |
| Enterobacteria phage I2-2 | 6.7 | 9 | 9 | 66.73 |
| Enterobacteria phage If1 | 8.5 | 10 | 10 | 80.18 |
| Enterobacteria phage Ike | 6.9 | 10 | 10 | 87.78 |
| Enterobacteria phage KU1 | 3.5 | 4 | 4 | 91.80 |
| Enterobacteria phage M13 | 6.4 | 10 | 10 | 83.28 |
| Enterobacteria phage Mu | 36.7 | 55 | 55 | 94.77 |
| Enterobacteria phage N15 | 46.4 | 60 | 60 | 90.90 |
| Enterobacteria phage P1 | 94.8 | 110 | 113 | 87.20 |
| Enterobacteria phage P2 | 33.6 | 43 | 43 | 92.28 |
| Enterobacteria phage P22 | 41.7 | 72 | 72 | 89.78 |
| Enterobacteria phage PRD1 | 14.9 | 22 | 22 | 82.15 |
| Enterobacteria phage RB43 | 180.5 | 292 | 293 | 93.80 |
| Enterobacteria phage RB49 | 164.0 | 274 | 274 | 93.76 |
| Enterobacteria phage RB69 | 167.6 | 273 | 275 | 93.83 |
| Enterobacteria phage S13 | 5.4 | 12 | 12 | 96.47 |
| Enterobacteria phage Sf6 | 39.0 | 66 | 68 | 90.30 |
| Enterobacteria phage SP6 | 43.8 | 52 | 52 | 91.04 |
| Enterobacteria phage T1 | 48.8 | 78 | 78 | 91.24 |
| Enterobacteria phage T3 | 38.2 | 55 | 55 | 91.15 |
| Enterobacteria phage T4 | 168.9 | 278 | 286 | 94.49 |
| Enterobacteria phage T5 | 121.8 | 162 | 187 | 81.31 |
| Enterobacteria phage T7 | 39.9 | 60 | 60 | 92.02 |
| Enterobacterio phage MS2 | 3.6 | 4 | 4 | 90.95 |
| Enterobacteriophage Qbeta | 4.2 | 4 | 4 | 95.30 |
| Guinea pig Chlamydia phage | 4.5 | 9 | 9 | 85.03 |
| Haemophilus phage HP1 | 32.4 | 42 | 43 | 92.33 |
| Haemophilus phage HP2 | 31.5 | 37 | 38 | 90.88 |
| Halorubrum phage HF2 | 77.7 | 114 | 120 | 88.36 |
| Lactobacillus bacteriophage phi adh | 43.8 | 63 | 63 | 90.68 |
| Lactobacillus casei bacteriophage A2 | 43.4 | 61 | 62 | 88.69 |
| Lactobacillus johnsonii prophage Lj928 | 38.4 | 50 | 51 | 90.46 |
| Lactobacillus johnsonii prophage Lj965 | 40.2 | 46 | 50 | 89.58 |
| Lactobacillus plantarum bacteriophage LP65 | 131.5 | 165 | 193 | 85.99 |
| Lactobacillus plantarum bacteriophage phiJL-1 | 36.7 | 46 | 47 | 85.23 |
| Lactococcus phage BK5-T | 40.0 | 63 | 63 | 90.87 |
| Lactococcus phage c2 | 22.2 | 39 | 39 | 92.60 |
| Lactococcus phage P335 | 36.6 | 49 | 49 | 92.91 |
| Lactococcus phage TP901-1 | 37.7 | 56 | 57 | 92.60 |
| Lactococcus phage ul36 | 36.8 | 58 | 58 | 88.77 |
| Listeria phage 2389 | 37.6 | 59 | 60 | 90.66 |
| Listonella pelagia phage phiHSIC | 38.0 | 47 | 47 | 87.65 |
| Methanobacterium phage psiM2 | 26.1 | 32 | 32 | 87.51 |
| Methanothermobacter wolfeii prophage psiM100 | 28.8 | 35 | 35 | 88.65 |
| Mycobacteria phage D29 | 49.1 | 79 | 84 | 91.43 |
| Mycobacteriophage PG1 | 69.0 | 100 | 100 | 94.25 |
| Mycobacterium phage Barnyard | 70.8 | 109 | 109 | 94.43 |
| Mycobacterium phage Bxb1 | 50.6 | 86 | 86 | 90.41 |
| Mycobacterium phage Bxz1 | 156.1 | 225 | 254 | 91.52 |
| Mycobacterium phage Bxz2 | 50.9 | 86 | 89 | 91.20 |
| Mycobacterium phage Che8 | 59.5 | 112 | 112 | 94.80 |
| Mycobacterium phage Che9c | 57.1 | 84 | 85 | 94.08 |
| Mycobacterium phage Che9d | 56.3 | 111 | 111 | 94.71 |
| Mycobacterium phage Cjw1 | 75.9 | 141 | 143 | 93.03 |
| Mycobacterium phage Corndog | 69.8 | 122 | 122 | 93.88 |
| Mycobacterium phage L5 | 52.3 | 85 | 88 | 88.13 |
| Mycobacterium phage Omega | 110.9 | 237 | 239 | 93.95 |
| Mycobacterium phage Rosebush | 67.5 | 90 | 90 | 95.26 |
| Mycobacterium phage TM4 | 52.8 | 89 | 89 | 91.91 |
| Mycoplasma arthritidis bacteriophage MAV1 | 15.6 | 15 | 15 | 90.23 |
| Propionibacterium phage phiB5 | 5.8 | 10 | 10 | 94.38 |
| Pseudoalteromonas phage PM2 | 10.1 | 22 | 22 | 92.82 |
| Pseudomonas aeruginosa bacteriophage PaP2 | 43.8 | 58 | 58 | 92.40 |
| Pseudomonas aeruginosa phage F116 | 65.2 | 70 | 70 | 92.91 |
| Pseudomonas aeruginosa phage PaP3 | 45.5 | 71 | 75 | 92.25 |
| Pseudomonas bacteriophage phi-13 | 13.7 | 13 | 13 | 84.44 |
| Pseudomonas phage D3 | 56.4 | 95 | 99 | 89.65 |
| Pseudomonas phage gh-1 | 37.4 | 42 | 42 | 93.26 |
| Pseudomonas phage Pf1 | 7.4 | 14 | 14 | 90.92 |
| Pseudomonas phage Pf3 | 5.8 | 9 | 9 | 92.78 |
| Pseudomonas phage phi-6 | 13.4 | 13 | 13 | 78.94 |
| Pseudomonas phage phiKZ | 280.3 | 306 | 313 | 87.27 |
| Pseudomonas phage PP7 | 3.6 | 4 | 4 | 95.62 |
| Ralstonia phage p12J | 7.1 | 10 | 10 | 77.19 |
| Roseophage SIO1 | 39.9 | 34 | 34 | 77.12 |
| Salmonella typhimurium bacteriophage ES18 | 46.9 | 79 | 79 | 91.68 |
| Salmonella typhimurium bacteriophage ST104 | 41.4 | 63 | 63 | 90.58 |
| Salmonella typhimurium bacteriophage ST64T | 40.7 | 65 | 66 | 90.77 |
| Salmonella typhimurium phage ST64B | 40.1 | 56 | 56 | 88.81 |
| Shigella flexneri bacteriophage V | 37.1 | 53 | 53 | 92.67 |
| Sinorhizobium meliloti phage PBC5 | 57.4 | 83 | 83 | 89.76 |
| Spiroplasma phage 1-C74 | 7.8 | 13 | 13 | 79.72 |
| Spiroplasma phage 1-R8A2B | 8.3 | 12 | 12 | 76.16 |
| Spiroplasma phage 4 | 4.4 | 9 | 9 | 82.29 |
| Staphylococcus aureus bacteriophage PVL | 41.4 | 62 | 62 | 89.79 |
| Staphylococcus aureus phage phi 11 | 43.6 | 53 | 53 | 79.90 |
| Staphylococcus aureus phage phi 12 | 45.0 | 49 | 49 | 79.65 |
| Staphylococcus aureus phage phi 13 | 42.7 | 49 | 49 | 78.07 |
| Staphylococcus aureus phage phiP68 | 18.2 | 22 | 22 | 92.25 |
| Staphylococcus aureus prophage phiPV83 | 45.6 | 65 | 65 | 87.22 |
| Staphylococcus aureus temperate phage phiSLT | 42.9 | 61 | 61 | 89.40 |
| Staphylococcus phage 44AHJD | 16.8 | 21 | 21 | 91.56 |
| Staphylococcus phage K | 127.4 | 115 | 119 | 78.68 |
| Staphylococcus phage phiN315 | 44.1 | 65 | 65 | 87.93 |
| Staphylococcus phage Twort | 130.7 | 195 | 196 | 88.06 |
| Streptococcus mitis phage SM1 | 34.7 | 56 | 56 | 91.43 |
| Streptococcus phage C1 | 16.7 | 20 | 20 | 87.15 |
| Streptococcus phage Cp-1 | 19.3 | 28 | 28 | 86.41 |
| Streptococcus pneumoniae bacteriophage MM1 | 40.2 | 53 | 53 | 94.37 |
| Streptococcus pyogenes phage 315.1 | 39.5 | 56 | 56 | 87.42 |
| Streptococcus pyogenes phage 315.2 | 41.1 | 60 | 60 | 88.66 |
| Streptococcus pyogenes phage 315.3 | 34.4 | 52 | 52 | 90.96 |
| Streptococcus pyogenes phage 315.4 | 41.8 | 64 | 64 | 88.42 |
| Streptococcus pyogenes phage 315.5 | 38.2 | 55 | 55 | 86.96 |
| Streptococcus pyogenes phage 315.6 | 40.0 | 51 | 51 | 90.64 |
| Streptococcus thermophilus bacteriophage 2972 | 34.7 | 44 | 44 | 93.35 |
| Streptococcus thermophilus bacteriophage 7201 | 35.5 | 46 | 46 | 93.21 |
| Streptococcus thermophilus bacteriophage DT1 | 34.8 | 45 | 45 | 89.18 |
| Streptococcus thermophilus bacteriophage Sfi11 | 39.8 | 53 | 53 | 93.06 |
| Streptococcus thermophilus bacteriophage Sfi19 | 37.4 | 45 | 45 | 88.93 |
| Streptococcus thermophilus bacteriophage Sfi21 | 40.7 | 50 | 50 | 89.36 |
| Streptococcus thermophilus temperate bacteriophage O1205 | 43.1 | 57 | 57 | 92.89 |
| Stx1 converting bacteriophage | 59.9 | 167 | 167 | 89.32 |
| Stx2 converting bacteriophage I | 61.8 | 166 | 169 | 89.44 |
| Stx2 converting bacteriophage II | 62.7 | 170 | 173 | 88.21 |
| Temperate phage phiNIH1.1 | 41.8 | 55 | 55 | 82.01 |
| Vibrio cholerae O139 fs1 phage | 6.3 | 15 | 15 | 82.08 |
| Vibrio cholerae phage KSF-1phi | 7.1 | 12 | 12 | 73.11 |
| Vibrio cholerae phage VGJphi | 7.5 | 13 | 13 | 83.07 |
| Vibrio harveyi bacteriophage VHML | 43.2 | 57 | 57 | 83.29 |
| Vibrio phage fs2 | 8.7 | 9 | 9 | 84.78 |
| Vibrio phage VP5 | 39.8 | 48 | 48 | 93.08 |
| Vibrio phage VSK | 6.9 | 14 | 14 | 85.34 |
| Vibriophage VP2 | 39.9 | 47 | 47 | 92.76 |
| Vibriophage VP4 | 39.5 | 21 | 21 | 68.82 |
| Vibriophage VpV262 | 46.0 | 67 | 68 | 91.79 |
| Xanthomonas campestris pv. pelargonii phage Xp15 | 55.8 | 84 | 84 | 90.28 |
| Xanthomonas oryzae bacteriophage Xp10 | 44.4 | 60 | 60 | 89.55 |
| Xanthomonas phage Cf1c | 7.3 | 9 | 9 | 80.32 |
| Yersinia pestis phage phiA1122 | 37.6 | 50 | 50 | 91.84 |
|  |  |  |  |  |
| **Mitochondria** |  |  |  |  |
| *Acanthaster brevispinus* mitochondrion | 16.3 | 13 | 37 |  |
| *Acanthaster planci* mitochondrion | 16.2 | 13 | 37 |  |
| *Acrochordus granulatus* mitochondrion | 17.6 | 13 | 37 | 64.00 |
| *Alectura lathami* mitochondrion | 16.7 | 13 | 37 | 68.00 |
| *Ambystoma andersoni* mitochondrion | 16.4 | 13 | 37 | 69.00 |
| *Ambystoma californiense* mitochondrion | 16.4 | 13 | 37 | 69.00 |
| *Ambystoma dumerilii* mitochondrion | 16.4 | 13 | 37 | 69.00 |
| *Andrias japonicus* mitochondrion | 16.3 | 13 | 37 | 69.00 |
| *Anisakis simplex* mitochondrion | 13.9 | 12 | 36 |  |
| *Aplysia californica* mitochondrion | 14.1 | 13 | 37 | 77.00 |
| *Arabidopsis thaliana* mitochondrion | 366.9 | 66 | 85 | 11.00 |
| *Aspergillus niger* mitochondrion | 31.1 | 16 | 43 |  |
| *Aspergillus tubingensis* mitochondrion | 33.7 | 16 | 43 |  |
| *Asterias amurensis* mitochondrion | 16.4 | 13 | 32 | 69.00 |
| *Astropecten polyacanthus* mitochondrion | 16.3 | 13 | 32 | 70.00 |
| *Bactrocera oleae* mitochondrion | 15.8 | 13 | 37 | 70.00 |
| *Balaenoptera edeni* mitochondrion | 16.4 | 13 | 37 |  |
| *Balaenoptera omurai* mitochondrion | 16.4 | 13 | 37 |  |
| *Balaenoptera physalus* mitochondrion | 16.4 | 13 | 37 | 69.00 |
| *Bipes canaliculatus* mitochondrion | 16.2 | 13 | 37 | 69.00 |
| *Boa constrictor* mitochondrion | 18.9 | 13 | 37 | 59.00 |
| *Bombina bombina* | 17.6 | 13 | 37 | 64.00 |
| *Bombyx mandarina* mitochondrion | 15.9 | 13 | 37 | 70.00 |
| *Bombyx mori* mitochondrion | 15.6 | 13 | 13 | 71.00 |
| *Bos grunniens* mitochondrion | 16.3 | 12 | 36 | 58.00 |
| *Bos indicus* mitochondrion | 16.3 | 13 | 36 | 69.00 |
| *Bos taurus* mitochondrion | 16.3 | 13 | 58 | 69.00 |
| *Branta canadensis* mitochondrion | 16.8 | 13 | 37 | 67.00 |
| *Buergeria buergeri* mitochondrion | 20.0 | 13 | 37 | 56.00 |
| *Cafeteria roenbergensis* mitochondrion | 43.1 | 34 | 58 | 82.00 |
| *Callinectes sapidus* mitochondrion | 16.3 | 13 | 37 | 68.00 |
| *Campanulotes bidentatus compar* mitochondrion | 14.8 | 13 | 37 |  |
| *Candida albicans SC5314* mitochondrion | 40.4 | 13 | 45 | 24.00 |
| *Candida glabrata* mitochondrion | 20.0 | 11 | 37 | 47.00 |
| *Candida metapsilosis* mitochondrion | 24.2 | 15 | 41 | 54.00 |
| *Candida orthopsilosis* mitochondrion | 22.5 | 15 | 41 | 56.00 |
| *Candida parapsilosis* mitochondrion | 32.7 | 20 | 46 |  |
| *Carassius auratus x Cyprinus carpio* mitochondrion | 16.6 | 13 | 37 |  |
| *Carassius auratus x Cyprinus carpio x Carassius cuvieri* mitochondrion | 16.6 | 13 | 37 |  |
| *Cathartes aura* mitochondrion | 16.8 | 13 | 37 | 67.00 |
| *Centruroides limpidus* mitochondrion | 14.5 | 13 | 36 | 74.00 |
| *Cercopithecus aethiops* mitochondrion | 16.4 | 13 | 37 | 69.00 |
| *Cervus elaphus* mitochondrion | 16.4 | 13 | 37 | 65.00 |
| *Cervus nippon yakushimae* mitochondrion | 16.4 | 13 | 37 | 68.00 |
| *Chauliodus sloani* mitochondrion | 17.8 | 13 | 37 | 64.00 |
| *Chirocentrus dorab* mitochondrion | 16.0 | 13 | 37 | 71.00 |
| *Chlorurus sordidus* mitochondrion | 16.7 | 13 | 37 | 68.00 |
| *Choloepus didactylus* mitochondrion | 16.5 | 13 | 37 | 68.00 |
| *Chondrus crispus* mitochondrion | 25.8 | 25 | 51 | 71.00 |
| *Chrysodidymus synuroideus* mitochondrion | 34.1 | 37 | 62 |  |
| *Ciona savignyi* mitochondrion | 14.7 | 12 | 38 | 74.00 |
| *Cobitis sinensis* mitochondrion | 16.6 | 13 | 37 | 68.00 |
| *Cordylus warreni* mitochondrion | 17.2 | 13 | 39 | 66.00 |
| *Coreoleuciscus splendidus* mitochondrion | 16.6 | 13 | 37 | 68.00 |
| *Crassostrea virginica* mitochondrion | 17.2 | 12 | 37 |  |
| *Cricetulus griseus* mitochondrion | 16.3 | 13 | 15 |  |
| *Crinipellis perniciosa* mitochondrion | 109.1 | 89 | 117 | 50.00 |
| *Crocidura russula* mitochondrion | 17.2 | 13 | 26 | 65.00 |
| *Cromeria nilotica* mitochondrion | 16.0 | 13 | 35 |  |
| *Cyanidioschyzon merolae* mitochondrion | 32.2 | 34 | 62 | 76.00 |
| *Cyanidioschyzon merolae* mitochondrion | 32.2 | 31 | 62 | 76.00 |
| *Cygnus columbianus* mitochondrion | 16.7 | 13 | 37 | 68.00 |
| *Cylindrophis ruffus* mitochondrion | 17.5 | 13 | 37 | 64.00 |
| *Cypselurus hiraii* mitochondrion | 16.5 | 13 | 37 | 69.00 |
| *Danio rerio* mitochondrion | 16.6 | 13 | 37 | 68.00 |
| *Dasyurus hallucatus* mitochondrion | 17.2 | 13 | 37 | 68.00 |
| *Denticeps clupeoides* mitochondrion | 17.2 | 13 | 37 | 65.00 |
| *Desmarestia viridis* mitochondrion | 39.0 | 39 | 68 | 69.00 |
| *Dictyostelium citrinum* mitochondrion | 58.6 | 33 | 46 | 68.00 |
| *Dictyota dichotoma* mitochondrion | 31.6 | 38 | 66 | 77.00 |
| *Diomedea melanophris* mitochondrion | 17.0 | 13 | 37 | 69.00 |
| *Doliolum nationalis* mitochondrion | 16.4 | 13 | 40 | 66.00 |
| *Drosophila melanogaster* mitochondrion | 19.5 | 13 | 37 | 57.00 |
| *Echinococcus multilocularis* mitochondrion | 13.7 | 12 | 36 | 73.00 |
| *Echymipera rufescens australis* mitochondrion | 16.6 | 13 | 37 | 68.00 |
| *Emmelichthys struhsakeri* mitochondrion | 16.5 | 13 | 37 | 68.00 |
| *Epidermophyton floccosum* mitochondrion | 30.9 | 64 | 51 | 67.00 |
| *Epigonichthys lucayanus* mitochondrion | 15.1 | 13 | 37 | 74.00 |
| *Epigonichthys maldivensis* mitochondrion | 15.0 | 13 | 37 | 75.00 |
| *Fejervarya limnocharis* mitochondrion | 17.7 | 13 | 38 | 63.00 |
| *Fucus vesiculosus* mitochondrion | 36.4 | 38 | 67 | 77.00 |
| *Gallus gallus gallus* mitochondrion | 16.8 | 13 | 37 | 67.00 |
| *Gallus lafayettei* mitochondrion | 16.8 | 13 | 37 | 67.00 |
| *Gallus sonneratii* mitochondrion | 16.8 | 13 | 37 | 67.00 |
| *Gavia stellata* mitochondrion | 17.6 | 13 | 37 | 67.00 |
| *Gegeneophis ramaswamii* mitochondrion | 15.9 | 13 | 36 | 70.00 |
| *Gekko gecko* mitochondrion | 16.4 | 13 | 37 | 68.00 |
| *Geochelone pardalis* mitochondrion | 19.4 | 13 | 37 | 54.00 |
| *Geothelphusa dehaani* mitochondrion | 18.2 | 13 | 38 | 61.00 |
| *Gonodactylus chiragra* mitochondrion | 16.3 | 13 | 37 | 68.00 |
| *Grasseichthys gabonensis* mitochondrion | 16.9 | 13 | 37 | 11.00 |
| *Gryllotalpa orientalis* mitochondrion | 15.5 | 13 | 37 | 71.00 |
| *Gymnocrinus richeri* mitochondrion | 16.0 | 13 | 37 | 71.00 |
| *Haematobia irritans irritans* mitochondrion | 16.1 | 13 | 37 | 69.00 |
| *Haliotis rubra* mitochondrion | 16.9 | 13 | 37 | 69.00 |
| *Halocynthia roretzi* mitochondrion | 14.8 | 12 | 37 | 72.00 |
| *Hanseniaspora uvarum* mitochondrion | 18.8 | 8 | 33 | 32.00 |
| *Harpiosquilla harpax* mitochondrion | 15.7 | 13 | 37 | 72.00 |
| *Hemibarbus labeo* mitochondrion | 16.6 | 13 | 37 | 68.00 |
| *Hemibarbus longirostris* mitochondrion | 16.6 | 13 | 37 | 68.00 |
| *Hemibarbus mylodon* mitochondrion | 16.6 | 13 | 37 | 68.00 |
| *Hexatrygon bickelli* mitochondrion | 17.5 | 13 | 37 | 65.00 |
| *Hyla chinensis* mitochondrion | 18.2 | 13 | 37 | 62.00 |
| *Ichthyophis bannanicus* mitochondrion | 16.0 | 13 | 37 | 70.00 |
| *Ilyanassa obsoleta* mitochondrion | 15.3 | 13 | 37 | 73.00 |
| *Indotestudo elongata* mitochondrion | 16.8 | 13 | 37 | 67.00 |
| *Japyx solifugus* mitochondrion | 15.8 | 13 | 37 | 70.00 |
| *Jenkinsia lamprotaenia* mitochondrion | 16.8 | 13 | 37 | 67.00 |
| *Kaloula pulchra* mitochondrion | 16.8 | 13 | 37 | 67.00 |
| *Kluyveromyces thermotolerans* mitochondrion | 23.6 | 11 | 38 | 41.00 |
| *Kneria sp. SL-2004* mitochondrion | 16.4 | 13 | 37 | 43.00 |
| *Larus dominicanus* mitochondrion | 16.7 | 13 | 37 | 68.00 |
| *Lates calcarifer* mitochondrion | 16.5 | 13 | 37 | 70.00 |
| *Latimeria menadoensis* mitochondrion | 16.5 | 13 | 37 | 65.00 |
| *Lecanicillium muscarium* mitochondrion | 24.5 | 15 | 42 | 58.00 |
| *Lepeophtheirus salmonis* mitochondrion | 15.4 | 12 | 36 | 70.00 |
| *Leptorhynchoides thecatus* mitochondrion | 13.9 | 12 | 38 | 74.00 |
| *Leptotrombidium akamushi* mitochondrion | 13.7 | 13 | 37 | 75.00 |
| *Leptotrombidium deliense* mitochondrion | 13.7 | 13 | 37 | 74.00 |
| *Leptotrombidium pallidum* mitochondrion | 16.8 | 13 | 38 | 61.00 |
| *Limnonectes fujianensis* mitochondrion | 17.7 | 13 | 37 | 42.00 |
| *Lipotes vexillifer* mitochondrion | 16.4 | 13 | 37 | 69.00 |
| *Lottia digitalis* mitochondrion | 26.8 | 13 | 35 | 43.00 |
| *Loxodonta africana* mitochondrion | 16.9 | 13 | 37 | 67.00 |
| *Luidia quinalia* mitochondrion | 16.5 | 13 | 32 | 69.00 |
| *Lyciasalamandra atifi* mitochondrion | 16.7 | 13 | 37 | 68.00 |
| *Lysiosquillina maculata* mitochondrion | 16.3 | 13 | 37 | 68.00 |
| *Macrobrachium rosenbergii* mitochondrion | 15.8 | 13 | 37 | 68.00 |
| *Malacochersus tornieri* mitochondrion | 19.2 | 13 | 38 | 59.00 |
| *Mammuthus primigenius* mitochondrion | 16.8 | 13 | 37 | 67.00 |
| *Manouria emys* mitochondrion | 16.5 | 13 | 37 | 74.00 |
| *Mantella madagascariensis* mitochondrion | 22.9 | 13 | 38 | 74.00 |
| *Marsupenaeus japonicus* mitochondrion | 16.0 | 13 | 37 | 69.00 |
| *Masturus lanceolatus* mitochondrion | 16.5 | 13 | 37 | 70.00 |
| *Melanogrammus aeglefinus* mitochondrion | 16.6 | 13 | 37 | 68.00 |
| *Menura novaehollandiae* mitochondrion | 17.8 | 13 | 37 | 69.00 |
| *Merlangius merlangus* mitochondrion | 16.6 | 13 | 37 | 69.00 |
| *Mesobuthus gibbosus* mitochondrion | 16.0 | 13 | 37 | 67.00 |
| *Microhyla heymonsi* mitochondrion | 16.7 | 13 | 37 | 67.00 |
| *Mola mola* mitochondrion | 16.5 | 13 | 37 | 67.00 |
| *Montastraea annularis* mitochondrion | 16.1 | 13 | 17 | 72.00 |
| *Montastraea faveolata* mitochondrion | 16.1 | 13 | 17 | 72.00 |
| *Mortierella verticillata* mitochondrion | 58.7 | 25 | 54 | 36.00 |
| *Mus musculus* mitochondrion | 16.3 | 13 | 57 | 69.00 |
| *Mytilus trossulus* mitochondrion | 18.7 | 12 | 38 | 73.00 |
| *Myxocyprinus asiaticus* mitochondrion | 16.6 | 13 | 37 | 68.00 |
| *Octopus ocellatus* mitochondrion | 16.0 | 13 | 37 | 70.00 |
| *Orbinia latreillii* mitochondrion | 15.6 | 13 | 37 | 68.00 |
| *Oreochromis mossambicus* mitochondrion | 16.6 | 13 | 37 | 69.00 |
| *Ornithodoros porcinus* mitochondrion | 14.4 | 13 | 37 | 75.00 |
| *Oryza sativa (indica cultivar-group)* mitochondrion | 491.5 | 54 | 93 | 68.00 |
| *Ovophis okinavensis* mitochondrion | 17.4 | 13 | 37 | 64.00 |
| *Pantholops hodgsonii* mitochondrion | 16.5 | 13 | 37 | 68.00 |
| *Paracoccidioides brasiliensis* mitochondrion | 71.3 | 17 | 44 | 68.00 |
| *Parakneria cameronensis* mitochondrion | 16.6 | 13 | 37 | 66.00 |
| *Paramecium aurelia* mitochondrion | 40.5 | 46 | 54 | 64.00 |
| *Paramesotriton hongkongensis* mitochondrion | 16.3 | 13 | 37 | 69.00 |
| *Pedinomonas minor* mitochondrion | 25.1 | 11 | 23 | 69.00 |
| *Periplaneta fuliginosa* mitochondrion | 15.0 | 13 | 37 | 74.00 |
| *Petrobius brevistylis* mitochondrion | 15.7 | 13 | 37 | 71.00 |
| *Phanogenia gracilis* mitochondrion | 15.9 | 13 | 37 | 72.00 |
| *Philaenus spumarius* mitochondrion | 16.3 | 13 | 37 | 67.00 |
| *Phocoena phocoena* mitochondrion | 16.4 | 13 | 26 | 59.00 |
| *Phractolaemus ansorgii* mitochondrion | 16.5 | 13 | 37 | 64.00 |
| *Physcomitrella patens* mitochondrion | 105.3 |  | 69 | 68.00 |
| *Phytophthora infestans* mitochondrion | 38.0 | 40 | 67 | 69.00 |
| *Placopecten magellanicus* mitochondrion | 32.1 | 12 | 48 | 35.00 |
| *Plasmodium simium* mitochondrion | 6.0 | 3 | 3 | 55.00 |
| *Plasmodium vivax* mitochondrion | 6.0 | 3 | 3 | 56.00 |
| *Podospora anserina* mitochondrion | 94.2 | 53 | 82 | 58.00 |
| *Polypedates megacephalus* mitochondrion | 16.5 | 11 | 35 | 56.00 |
| *Porichthys myriaster* mitochondrion | 18.9 | 13 | 37 | 60.00 |
| *Pseudocarcinus gigas* mitochondrion | 15.5 | 13 | 37 | 67.00 |
| *Pterodroma brevirostris* mitochondrion | 16.4 | 13 | 37 | 67.00 |
| *Pteronarcys princeps* mitochondrion | 16.0 | 13 | 37 | 70.00 |
| *Pyrocoelia rufa* mitochondrion | 17.7 | 13 | 37 | 62.00 |
| *Python regius* mitochondrion | 17.2 | 13 | 37 | 65.00 |
| *Raja porosa* mitochondrion | 17.0 | 13 | 37 | 67.00 |
| *Rangifer tarandus* mitochondrion | 16.4 | 13 | 37 | 69.00 |
| *Ranzania laevis* mitochondrion | 16.5 | 13 | 37 | 69.00 |
| *Rattus norvegicus* mitochondrion | 16.3 | 13 | 37 | 69.00 |
| *Rattus norvegicus strain BN/SsNHsdMCW* mitochondrion | 16.3 | 13 | 37 | 69.00 |
| *Reclinomonas americana* mitochondrion | 69.0 | 67 | 97 | 80.00 |
| *Rhacophorus schlegelii* mitochondrion | 21.4 | 13 | 37 | 52.00 |
| *Rhizopus oryzae* mitochondrion | 54.2 | 24 | 51 | 38.00 |
| *Rhodeus uyekii* mitochondrion | 16.8 | 13 | 37 | 69.00 |
| *Roboastra europaea* mitochondrion | 14.5 | 13 | 37 | 75.00 |
| *Rousettus aegyptiacus* mitochondrion | 16.7 | 13 | 37 | 67.00 |
| *Saccharomyces cerevisiae* mitochondrion | 85.8 | 19 | 46 | 23.00 |
| *Saccoglossus kowalevskii* mitochondrion | 17.0 | 13 | 37 | 66.00 |
| *Salanx ariakensis* mitochondrion | 16.6 | 13 | 37 | 68.00 |
| *Scenedesmus obliquus* mitochondrion | 42.8 | 20 | 53 | 38.00 |
| *Schizosaccharomyces pombe* mitochondrion | 19.4 | 10 | 37 | 50.00 |
| *Scleropages formosus* mitochondrion | 16.7 | 13 | 37 | 68.00 |
| *Sclerophasma paresisensis* mitochondrion | 15.5 | 13 | 37 | 71.00 |
| *Scolecomorphus vittatus* mitochondrion | 16.0 | 13 | 37 | 70.00 |
| *Scomber scombrus* mitochondrion | 16.6 | 13 | 37 | 68.00 |
| *Sebastes schlegeli* mitochondrion | 16.5 | 13 | 37 | 69.00 |
| *Sepia officinalis* mitochondrion | 16.2 | 13 | 37 | 65.00 |
| *Sepioteuthis lessoniana* mitochondrion | 16.6 | 13 | 37 | 69.00 |
| *Siphonops annulatus* mitochondrion | 16.2 | 13 | 37 | 69.00 |
| *Sminthopsis crassicaudata* mitochondrion | 17.4 | 13 | 37 | 69.00 |
| *Smittium culisetae* mitochondrion | 58.7 | 31 | 61 | 43.00 |
| *Sorex unguiculatus* mitochondrion | 17.1 | 13 | 37 | 66.00 |
| *Spadella cephaloptera* mitochondrion | 11.9 | 11 | 13 | 81.00 |
| *Spizaetus alboniger* mitochondrion | 18.0 | 13 | 37 | 63.00 |
| *Spizaetus nipalensis* mitochondrion | 17.7 | 13 | 37 | 64.00 |
| *Squilla empusa* mitochondrion | 15.8 | 13 | 37 | 70.00 |
| *Steinernema carpocapsae* mitochondrion | 13.9 | 12 | 36 | 73.00 |
| *Sundasalanx mekongensis* mitochondrion | 16.7 | 13 | 37 | 68.00 |
| *Sus scrofa* mitochondrion | 16.6 | 13 | 37 | 68.00 |
| *Synodus variegatus* mitochondrion | 16.5 | 13 | 37 | 69.00 |
| *Taenia asiatica* mitochondrion | 13.7 | 12 | 36 | 73.00 |
| *Taeniopygia guttata* mitochondrion | 16.9 | 13 | 37 | 81.00 |
| *Tamolanica tamolana* mitochondrion | 16.1 | 13 | 37 | 69.00 |
| *Teratoscincus keyserlingii* mitochondrion | 17.2 | 13 | 37 | 64.00 |
| *Testudo graeca* mitochondrion | 19.3 | 13 | 37 | 74.00 |
| *Testudo horsfieldii* mitochondrion | 17.1 | 13 | 37 | 66.00 |
| *Testudo kleinmanni* mitochondrion | 17.7 | 13 | 37 | 64.00 |
| *Testudo marginata* mitochondrion | 19.5 | 13 | 37 | 58.00 |
| *Tetrahymena pyriformis* mitochondrion | 47.3 | 44 | 58 | 62.00 |
| *Tetraodon nigroviridis* mitochondrion | 16.5 | 13 | 37 | 69.00 |
| *Thalassiosira pseudonana* mitochondrion | 43.8 | 35 | 62 | 64.00 |
| *Thrips imaginis* mitochondrion | 15.4 | 13 | 38 | 70.00 |
| *Triticum aestivum* mitochondrion | 452.5 | 39 | 73 | 7.00 |
| *Verasper variegatus* mitochondrion | 17.3 | 13 | 37 | 24.00 |
| *Verasper variegatus* mitochondrion | 17.0 | 13 | 37 | 67.00 |
| *Verasper variegatus* mitochondrion | 14.8 | 13 | 39 | 74.00 |
| *Volemys kikuchii* mitochondrion | 16.3 | 13 | 37 | 69.00 |
| *Watasenia scintillans* mitochondrion | 20.1 | 18 | 43 |  |
| *Xenopeltis unicolor* mitochondrion | 18.9 | 13 | 37 | 59.00 |
| *Yarrowia lipolytica* mitochondrion | 48.0 | 24 | 53 | 45.00 |
|  |  |  |  |  |
| **Chloroplasts** |  |  |  |  |
| *Acorus calamus* plastid | 153.8 | 84 | 130 | 49.00 |
| *Adiantum capillus-v*eneris chloroplast | 151.0 | 87 | 130 |  |
| *Anthoceros formosae* chloroplast | 161.0 | 90 | 135 |  |
| *Arabidopsis thaliana* chloroplast | 154.0 | 87 | 132 |  |
| *Atropa belladonna* chloroplast | 156.7 | 87 | 132 | 51.00 |
| *Calycanthus floridus* *var. glaucus* chloroplast | 153.0 | 88 | 134 |  |
| *Chaetosphaeridium globosum* chloroplast | 131.0 | 98 | 141 |  |
| *Cucumis sativus* chloroplast | 155.3 | 85 | 131 | 49.00 |
| *Cyanophora paradoxa* chloroplast | 135.6 | 149 | 192 |  |
| *Emiliania huxleyi* chloroplast | 105.3 | 119 | 155 | 76.00 |
| *Euglena gracilis* chloroplast | 143.0 | 66 | 114 | 66.00 |
| *Glycine max* chloroplast | 152.0 |  | 128 | 48.00 |
| *Gossypium hirsutum* chloroplast | 160.0 |  | 131 | 44.00 |
| *Gracilaria tenuistipitata* *var. liui* chloroplast | 183.9 | 203 | 238 | 80.00 |
| *Lactuca sativa* chloroplast | 152.7 | 84 | 128 | 47.00 |
| *Lycopersicon esculentum* chloroplast | 155.0 | 86 | 132 | 54.00 |
| *Nicotiana tabacum* plastid | 155.9 | 101 | 146 | 49.00 |
| *Nicotiana tomentosiformis* chloroplast | 155.7 | 109 | 155 | 54.00 |
| *Phalaenopsis aphrodite* *subsp. formosana* chloroplast | 149.0 | 97 | 141 | 44.00 |
| *Porphyra yezoensis* chloroplast | 192.0 | 209 | 264 | 51.00 |
| *Psilotum nudum* chloroplast | 138.8 | 101 | 150 | 55.00 |
| *Solanum bulbocastanum* chloroplast | 155.0 |  | 142 | 80.00 |
| *Spinacia oleracea* plastid | 151.0 | 98 | 143 | 48.00 |
| *Theileria parva* strain Muguga apicoplast | 39.6 | 44 | 70 | 66.00 |
| *Toxoplasma gondii* apicoplast chloroplast | 35.0 | 26 | 63 | 52.00 |

*Total gene number equal to protein-coding gene number plus non-protein coding RNA gene number. Sources of data are as shown in the reference list below.

**Most gene-coding percentage data were retrieved from the percent of DNA coding number of bases over total number of bases from the Integrated Microbial Genomes (IMG) database in the Department of Energy Joint Genome Institute (JGI) website (http://img.jgi.doe.gov/cgi-bin/pub/main.cgi). Note that JGI’s estimated DNA coding number of bases includes bases from introns and other untranslated regions in addition to coding regions, thus the gene-coding percentages were overestimated for most eukaryotes. Therefore, for eukaryotic gene-coding percentages only data retrieved from peer-reviewed literature (in parentheses) were used in our analyses.

References:

1. Markowitz VM, Szeto E, Palaniappan K, Grechkin Y, Chu K, et al. (2007) The integrated microbial genomes (IMG) system in 2007: data content and analysis tool extensions. Nucl Acids Res: gkm846.
2. Reference Sequence (RefSeq) collection in National Center for Biotechnology Information (NCBI, http://www.ncbi.nlm.nih.gov).
3. Xu L, Chen H, Hu X, Zhang R, Zhang Z, et al. (2006) Average gene length is highly conserved in prokaryotes and eukaryotes and diverges only between the two kingdoms. Mol Biol Evol 23: 1107-1108.
4. Nierman WC, Pain A, Anderson MJ, Wortman JR, Kim HS, et al. (2005) Genomic sequence of the pathogenic and allergenic filamentous fungus *Aspergillus fumigatus*. 438: 1151-1156.
5. Archibald, JM (2007) Nucleomorph genomes: structure, function, origin and evolution. BioEssays 29: 392-402.
6. Biology analysis group, Xia Q, Zhou Z, Lu C, Cheng D, et al. (2004) A draft sequence for the genome of the domesticated silkworm (Bombyx mori). Science 306: 1937-1940.
7. Merchant SS, Prochnik SE, Vallon O, Harris EH, Karpowicz SJ, et al. (2007) The *Chlamydomonas* genome reveals the evolution of key animal and plant functions. Science 318: 245-250.
8. Lane CE, van den Heuvel K, Kozera C, Curtis BA, Parsons BJ, et al. (2007) Nucleomorph genome of *Hemiselmis andersenii* reveals complete intron loss and compaction as a driver of protein structure and function. Proc. Natl. Acad. Sci. USA 104: 19908-19913.
9. Matsuzaki M, Misumi O, Shin-i T, Maruyama S, Takahara M, et al. (2004) Genome sequence of the ultrasmall unicellular red alga *Cyanidioschyzon merolae* 10D. Nature 428: 653-657.
10. International Human Genome Sequencing Consortium. (2004) Finishing the euchromatic sequence of the human genome. Nature 431: 931-945.
11. Derelle E, Ferraz C, Rombauts S, Rouzé P, Worden AZ, et al. (2006) Genome analysis of the smallest free-living eukaryote *Ostreococcus tauri* unveils many unique features. Proc. Natl. Acad. Sci. USA 103: 11647-11652.
12. Aury JM, Jaillon O, Duret L, Noel B, Jubin C, et al. (2006) Global trends of whole-genome duplications revealed by the ciliate *Paramecium tetraurelia*. Nature 444: 171-178.
13. Bowler C, Allen AE, Badger JH, Grimwood J, Jabbari K, et al. (2008) The *Phaeodactylum* genome reveals the evolutionary history of diatom genomes. Nature 456: 239-244.
14. Armbrust EV, Berges JA, Bowler C, Green BR, Martinez D, et al. (2004) The genome of the diatom *Thalassiosira pseudonana*: ecology, evolution, and metabolism. Science 306: 79-86.
15. Eisen JA, Coyne RS, Wu M, Wu D, Thiagarajan M, et al. (2006) Macronuclear genome sequence of the ciliate *Tetrahymena thermophila*, a model eukaryote. PLoS Biol 4: e286.
